# A chemical analysis of the *Pelargonium* species: *P*. *odoratissimum*, *P*. *graveolens*, and *P*. *zonale* identifies secondary metabolites with activity against gram-positive bacteria with multidrug-resistance

**DOI:** 10.1371/journal.pone.0306637

**Published:** 2024-07-10

**Authors:** Diana Celi, Evelyn Quiroz, Andrea Beltrán-Noboa, António Machado, Eduardo Tejera, Paulina Fernandez-Soto

**Affiliations:** 1 Facultad de Ingeniería y Ciencias Aplicadas, Universidad de Las Américas (UDLA), Quito, Ecuador; 2 Laboratorios de Investigación, Universidad de Las Américas (UDLA), Quito, Ecuador; 3 Grupo de Bioquimioinformática, Universidad de Las Américas (UDLA), Quito, Ecuador; 4 Departamento de Química Analítica, Universidad del País Vasco/Euskal Herriko Unibertsitatea (UPV/EHU), Bilbao, Spain; 5 Laboratorio de Bacteriología, Instituto de Microbiología, Colegio de Ciencias Biológicas y Ambientales COCIBA, Universidad San Francisco de Quito USFQ, Quito, Ecuador; 6 Facultad de Ciencias de la Salud, Universidad de Las Américas (UDLA), Quito, Ecuador; Universidad Autonoma de Chihuahua, MEXICO

## Abstract

The *Pelargonium* genus encompasses around 280 species, most of which are used for medicinal purposes. While *P*. *graveolens*, *P*. *odoratissimum*, and *P*. *zonale* are known to exhibit antimicrobial activity, there is an evident absence of studies evaluating all three species to understand their chemical differences and biological effects. Through the analysis of the hydroalcoholic extracts of *P*. *graveolens*, *P*. *odoratissimum*, and *P*. *zonale*, using HPLC-DAD-MS/MS, quercetin and kaempferol derivatives were identified in these three species. Conversely, gallotannins and anthocyanins were uniquely detected in *P*. *zonale*. *P*. *graveolens* stood out due to the various types of myricetin derivatives that were not detected in *P*. *odoratissimum* and *P*. *zonale* extracts. Evaluation of their biological activities revealed that *P*. *zonale* displayed superior antibacterial and antibiofilm activities in comparison to the other two species. The antibacterial efficacy of *P*. *zonale* was observed towards the clinically relevant strains of *Staphylococcus aureus* ATCC 25923, Methicillin-resistant *Staphylococcus aureus* (MRSA) 333, *Enterococcus faecalis* ATCC 29212, and the Vancomycin-resistant *E*. *faecalis* INSPI 032. Fractionation analysis of *P*. *zonale* suggested that the antibacterial activity attributed to this plant is due to the presence of quercetin derivatives and kaempferol and its derivatives, alongside their synergistic interaction with gallotannins and anthocyanins. Lastly, the three *Pelargonium* species exhibited notable antioxidant activity, which may be attributed to their high content of total phenolic compounds.

## Introduction

The *Pelargonium* genus is the second largest genus of the Geraniaceae family, containing around 280 species. Most of these are scented, widely used in perfume and cosmetic industries, and important for ornamental and medicinal purposes [1,2]. Among various *Pelargonium* species, *P*. *graveolens*, *P*. *odoratissimum*, and *P*. *zonale* have been of particular interest for their medicinal applications.

*P*. *graveolens* is the most studied plant within the *Pelargonium* genus. *P*. *graveolens* decoction and infusion preparations [3], essential oils [4,5], and organic and aqueous extracts [5–7] have shown antioxidant, antifungal, antiviral, anti-inflammatory, hepatoprotective and/or antimicrobial activities. Likewise, *P*. *zonale*’s biological activities are described as anticancer, antifungal, and antiviral when using ethanolic extracts [8,9], and antibacterial activity towards clinical strains has been reported in *P*. *zonale* essential oils [10]. In contrast to *P*. *graveolens* and *P*. *zonale*, little is known about *P*. *odoratissimum*’s biological activities, with few reports on its antioxidant activity [11,12] and its antimicrobial activity using essential oils [13,14] and methanolic extracts [12].

Studies employing hydroalcoholic extracts of *P*. *graveolens* [5–7], *P*. *zonale* [8,9], and *P*. *odoratissimum* [12] have revealed their antibacterial and/or antifungal activity against clinically relevant strains. However, none of these studies have compared the biological activities of these three species together, precluding an understanding of their potential similarities or differences. Likewise, reports of their chemical profiles have predominantly utilised crude extracts or essential oils preparations, with a lack of studies showing fractionation analyses [10,12,15–17]. Herein, we report the chemical profiles of the hydroalcoholic extracts of *P*. *graveolens*, *P*. *zonale*, and *P*. *odoratissimum*, along with an exploration of their biological activities as an antioxidant, antibacterial, and antibiofilm. Upon analysis of their antibacterial activity, fractionation of *P*. *zonale* was conducted, revealing secondary metabolites that may be responsible for its biological activities.

## Materials and methods

### Plant extracts preparation

Fresh plants of *P*. *odoratissimum*, *P*. *graveolens*, and *P*. *zonale* were purchased in October 2022 from two traditional markets of the Quito Metropolitan District in Ecuador: Santa Clara and Iñaquito. Identification and validation of the plants were carried out by specialists from the Botanical Garden of Quito, Ecuador. Table 1 shows the common Spanish names, scientific names, and parts of the plants used in this study. The parts of the selected plants were mixed to prepare one extract for each species. The only extract containing flowers in the preparation was *P*. *zonale*, as it is commonly sold with flowers to prepare infusions or drinks. Therefore, we decided to keep the flowers in the preparation of *P*. *zonale* extract to maintain the traditional medicinal uses.

**Table 1:** Plants and parts of the plants used in this study.

N	Common name	Scientific name	Part of the plant
1	Malva esencia or esencia de rosa	*Pelargonium graveolens* L’Hér.ex. Aiton	Leaf and stalk
2	Malva olorosa	*Pelargonium odoratissimum* (L.) L´Hér.	Leaf and stalk
3	Geranio or geranio rojo	*Pelargonium zonale* (L.) L´Hér.	Leaf, stalk, and flowers

The plant extracts were prepared according to our previous studies [12,18,19]. Initially, 0.5 kg of fresh plants were dried and ground to fineness using liquid nitrogen and stored at -80°C. To prepare the methanolic extract, 2 g of the ground sample was extracted with 20 mL of a methanol-water solution (80:20, v/v) (methanol 99% v/v; Sigma-Aldrich, St. Louis, MO, USA) and stirred for 2 h in the dark at room temperature (around 21°C). The samples were then centrifuged twice at 1500 rpm for 10 min and filtered through a cellulose filter paper (0.2 μm; Whatman, Merck & Co., Inc, USA). The supernatant was concentrated under vacuum using a Büchi Rotavapor R-210 set (BÜCHI Labortechnik, Switzerland) at 30°C in a water bath and cooling water at 0°C until most of the solvent was removed. The concentrate was then dried in a miVAC centrifugal concentrator (GeneVacTM, Thermo Fisher Scientific, Sweden) at 30°C for 5 h. Dried extracts were stored at -80°C for further use. Stock solutions were prepared by resuspension of the dry sample in 1 mL of methanol-water solution (80:20, v/v).

### Phytochemical screening

The hydroalcoholic extracts of *P*. *odoratissimum*, *P*. *graveolens*, and *P*. *zonale* were tested for the presence of phenolics, flavonoids, tannins, anthocyanins, and amino acids. Total phenolic content (TPC) was determined using the Folin-Ciocalteu colourimetric method [20]. For this, an aliquot of 100 mL of the resuspended extract, standard or blank, was mixed with 500 mL of 1:10 (v/v) Folin-Ciocalteu reagent (2N, Sigma-Aldrich, USA). The mixture was incubated for 5 min, and then 400 mL of 0.7 M sodium carbonate solution (PanReac AppliChem, Spain) was added and incubated for 2 h at room temperature in a dark room. The absorbance was then measured in a plate reader (Synergy HT, Bio-Tek Instruments, Inc., USA) at 760 nm against a blank of Milli-Q water. Gallic acid (Loba Chemie Pvt. Ltd., India) was used to construct the standard curve (0.5–1.2 mM). Results were expressed in milligrams of gallic acid equivalents (GAE) per gram of dry extract (DE) as mg GAE per g DE.

Total flavonoid content (TFC) was determined by the aluminium chloride colourimetric method [21]. For this, 80 mL of the suspended extract, standard or blank, was mixed with 400 mL of Milli-Q water and then added 24 mL of 5% sodium nitrite solution (Sigma-Aldrich, USA). After 6 min, 48 mL of a methanolic solution of aluminium chloride hexahydrate (10%, Loba Chemie Pvt. Ltd., India) was added to the mixture and incubated for a further 5 min. The mixture was then treated with 160 mL of 1 M sodium hydroxide solution and 88 mL of Milli-Q water and incubated for 15 min at room temperature in a dark room. The final mixture was analysed by measuring the absorbance in a plate reader at 510 nm with Milli-Q water as a blank. Catechin (Cayman Chemical, USA) was used to construct the standard curve (0.0625 mM to 1.0 mM), and results were expressed as milligrams of catechin equivalents (CE) per gram of dry extract (DE) (mg CE/g DE).

The total tannin content (TTA) was determined according to the method outlined in ISO 9648:19880 “Sorghum—Determination of tannin content”, available at https://www.iso.org/standard/17487.html (International Organization for Standardization, 1988). For this, an aliquot of 100 μL of the extract, standard or blank, was mixed with 100 μL of N-N-dimethylformamide 75% reagent (Sigma-Aldrich, USA). The mixture was incubated for 20 min at room temperature with stirring and then centrifuged at 3000 (RCF) for 10 min. The supernatant was collected and mixed with either solution A or B. For solution A, 75 μL of supernatant was mixed with 450 μL distilled water, and 75 μL ammonia solution was then stirred for a few seconds. For solution B, 75 μL of supernatant was mixed with 375 μL of distilled water and 75 μL of ferric ammonium citrate, stirred for a few seconds, and finally mixed with 75 μL of ammonia solution. The mixtures were incubated for 10 min in a dark room. The absorbance was then measured using a plate reader at 525 nm against a blank of distilled water and the solutions A or B. The TTA is the difference between absorbances B-A. The results were expressed as milligrams of tannic acid (TAEq) per gram of dry extract (DE) (TAEq/g DE).

Total monomeric anthocyanin pigment content (ACY) was determined using the pH differential method [22]. For this, a buffer pH of 1.0 (potassium chloride, 0.025M) and a buffer pH of 4.5 (sodium acetate, 0.4M) were used. A stock of pelargonidin solution (Sigma-Aldrich, USA) was used to generate a standard curve (10 mg to 150 mg). For each sample, 100 μL of standard or extract was added to either 900 μL of buffer pH 1.0 or 900 μL of buffer pH 4.5. For the blanks, 100 μL of distilled water was added to 900 μL of buffer pH 1.0 or buffer pH 4.5. The absorbance was read at 500 and 700 nm for both buffer solutions. Anthocyanin content was expressed as mg pelargonidin equivalents (mg PgEq) per mg of dry extract (DE) (mg PgEq/g DE).

The modified colourimetric ninhydrin method determined the total free amino acid content (FAA) [23]. For this, a working solution was prepared with a mixture of ninhydrin (Loba Chemie Pvt. Ltd., India), ethanol 99.5%, acetic acid, and CdCl_2_ (Sigma-Aldrich, USA). L- leucine (Sigma-Aldrich, USA) solution was used to generate a standard curve (2.4 mg/L to 60 mg/L). Then, 400 μL standard or extract was mixed with 800 μL of working solution. For the blank, 400 μL of distilled water and 800 μL of working solution were used. The solutions were heated in a water bath (Memmert-Ghb14) at 84°C for 5 min, and after cooling in a cold block, the absorbance was read at 507 nm using a plate reader. The results were expressed as mg of Leucine equivalents (LEeq) per g of dry extract (DE) (LEeq/g DE).

### Antioxidant activity analysis

The total antioxidant capacity (TAC) was analysed by two methods: the ferric reducing antioxidant power (FRAP) assay [24] and the 2,2-diphenyl-1-picrylhydrazyl (DPPH) free radical method [25]. For both assays, Trolox (Sigma-Aldrich, USA) was used to generate a standard curve (50 to 500 μM), and results were expressed as μmol Trolox equivalents (TEq) per gram of dry extract (μmol TEq/g DE). For the FRAP assay, a fresh FRAP reagent was prepared daily by combining sodium acetate trihydrate buffer (300 mM, Fisher Chemical, USA), 2,4,6-tri(2-pyridyl)-1,3,5-triazine (TPTZ) (10 mM in 40 mM HCl, Sigma-Aldrich, USA) and FeCl_3_6H_2_O solution (20 mM, Loba Chemie Pvt. Ltd, India) in a ratio of 10:1:1 and incubated at room temperature for 2 h. Then, 20 μL of extract, standard or blank, was mixed with 180 μL of FRAP solution, and the absorbance of the resulting mixture was measured at 593 nm using a plate reader.

For the DPPH assay, 50 μL of the extract was mixed with 400 μL of DPPH solution (0.2 mM, Alpha Aesar, USA) in absolute methanol and 550 μL of ethanol (70%). Two blank samples, B1 and B2, were prepared as follows: B1 consisted of 400 μL of DPPH solution, 50 μL of absolute methanol, and 550 μL of ethanol (70%), while B2 was prepared with 450 μL of absolute methanol, and 550 μL of ethanol (70%). After incubation for 15 min at room temperature, the absorbance of the extracts was determined at 517 nm using a plate reader. DPPH scavenging activity was calculated according to previous studies [12,26].

### HPLC-MS/MS analysis of *P*. *odoratissimum*, *P*. *graveolens*, and *P*. *zonale*

A total of 100 mg of dry extract was resuspended in 1 mL of methanol-water solution (80:20, v/v), filtered, and used for HPLC-MS/MS analysis. The system used for HPLC analysis was a Vanquish (Thermo Fisher Scientific, Massachusetts, USA) equipped with a dual pump and diode array detection (DAD) coupled to an LTQ-XL (Thermo Fisher Scientific, Massachusetts, USA) and controlled by Xcalibur software. Separation was performed on an Accucore Vanquish C18 column (1.5 μm, 100 × 2.1 mm; Merck KGaA, Darmstadt, Germany) at a constant temperature of 35°C. The mobile phase consisted of a solution of 0.1% formic acid (A) and acetonitrile (B). The elution gradient was set as follows: 2% B, 0–4 min; 4% B, 4–22 min; 40% B, 22–32 min; 70% B, 32–40 min; 2% B, 40–45 min, and the column was re-balanced to the initial solvent conditions. The injection volume was 50 μL, and the flow rate was set to 0.2 mL/min. The DAD performed double line detection at 220, 280, 330, and 370 nm, while the mass spectrometer (MS) was operated in the positive ion mode. Recorded spectra ranged from 50 to 2000 *m/z*. In positive ionisation mode, the electrospray ionisation (ESI) conditions were capillary temperature of 275°C, source voltage of 4.5 kV, capillary voltage of 18 V, and tube lens of 70. The tentative identification of the parent ions in positive ion mode was based on the fragmentation patterns. The patterns were compared with the MzCloud database (https://www.mzcloud.org/), and those with a match score of more than 70% were selected. In addition, fragmentation patterns were identified by a literature search and the GNPS database [27]. Retention time was also considered for the tentative identification in cases where ions with the same fragmentation pattern were detected in two or three *Pelargonium* species.

### Fractioning and chemical characterisation of *P*. *zonale*

The fractioning process of *P*. *zonale* extract was performed following the procedure described by Grkovic et al. [28] with some modifications. Briefly, 200 mg of dried extract was resuspended in 2 mL of a methanol-water solution (80:20, v/v) in a cotton swab (23 mm x 80 mm) stored in a solid phase extraction (SPE) column Bond Elut C18 (Agilent, USA), followed by a lyophilisation process to remove the solvent. The SPE column was equilibrated with 10 mL of methanol-water (5:95, v/v). The following eluents were used to collect the fractions: a) methanol-water (5:95, v/v), b) methanol-water solution (20:80, v/v), c) methanol-water solution (40:60, v/v), d) methanol-water solution (60:40, v/v), e) methanol-water solution (80:20, v/v) f) methanol and f) acetonitrile-methanol (50:50, v/v). Of each eluent, 2 mL was added to the column, and 1 mL was collected separately in 2 mL Eppendorf tubes that had been previously weighed. A total of 14 fractions were obtained, which were ultra-cooled to -80°C and then lyophilised. After lyophilisation, the fractions were weighed and re-suspended in a methanol-water solution (20:80, v/v) for drug susceptibility assays.

The HPLC-MS/MS analysis from fractions 1 to 10 was carried out with ZORBAX Eclipse Plus C18 column (5 μm, 150 × 4.6 mm; Agilen USA). The mobile phase consisted of 0.1% formic acid (A) and acetonitrile (B) solutions. The elution gradient was set up in the following way: 20% B, 0–5 min; 25% B, 5–10 min; 40% B, 10–20 min; 55% B, 20–30 min; 65% B, 30–50 min and the column was re-balanced to the initial solvent conditions. The mass spectrometry conditions and further analysis of identification are the same as explained above for the three species.

### Bacterial strains, cells, media, and growth conditions

The bacterial and yeast strains *Enterococcus faecium* ATCC 27270, *Enterococcus faecalis* ATCC 29212, *Staphylococcus aureus* ATCC 25923, *Acinetobacter baumannii* ATCC 19606, *Pseudomonas aeruginosa* ATCC 27853, *Enterobacter cloacae* ATCC 23355, *Escherichia coli* ATCC25922, *Candida albicans* ATCC 10231, and *Candida tropicalis* ATCC 1369 were obtained from the American Type Culture Collection (ATCC) [12]. The strains Methicillin-resistant *S*. *aureus* 333 previously isolated from nasal samples [29] were obtained from the Research Laboratories of the Universidad de Las Américas (UDLA); while Vancomycin-resistant *E*. *faecalis* INSPI 032 were donated by the National Institute for Research in Public Health (INSPI) (http://www.investigacionsalud.gob.ec/webs/ram/evaluacion-calidad/) to the Institute of Microbiology of the Universidad San Francisco de Quito (IM-USFQ). Bacterial strains were initially grown on Nutrient Agar (NA) at 37°C for 24 h and yeast strains on Sabouraud Dextrose Agar (SDA, both from Becton, Dickinson and Company, USA) at 35°C for 48 h. Raw 264.7 murine macrophages (ATCC) were cultured in Dulbecco′s Modified Eagle′s Medium (DMEM, Sigma-Aldrich, USA) containing 25 mM Glucose (4500 mg/L), 4 mM L-glutamine, 1 mM sodium pyruvate, 1500 mg/L sodium bicarbonate and supplemented with 10% heat inactivated fetal bovine serum (FBS, Invitrogen) at 37°C in a 5% CO_2_.

### Screening of antibacterial activity

The antimicrobial activity of *P*. *graveolens*, *P*. *odoratissimum*, and *P*. *zonale* methanolic extracts was initially assessed using a microdilution assay. Briefly, single bacterial colonies were selected to prepare an inoculum corresponding to 1.5 × 10^8^ colony-forming units (CFU)/mL using a 0.5 McFarland standard turbidity. Bacterial suspensions were adjusted to a final microorganism density of 5 × 10^5^ CFU/mL. For yeast, single colonies were selected to prepare an inoculum of an optical density between 0.12 to 0.15 at 530 nm, corresponding to 1 × 10^6^–5 × 10^6^ CFU/mL. Yeast suspensions were adjusted to a final microorganism density of 2 × 10^3^ CFU/mL. Working solutions of crude plant extracts were prepared by diluting the stock solutions with Mueller Hinton Broth (MHB; Becton, Dickinson and Company, USA) to a final concentration of 2000 μg/mL. A 96-well U-shaped plate (Tecan Group Ltd., Switzerland) was filled with 100 μL of working solution and allowed methanol evaporation for 30 min. Then, 100 μL of bacterial or yeast suspension was added to each well. The microplate was incubated at 37°C between 16 to 20 h for bacteria and at 35°C for 48 h for yeast. The final concentration of plant crude extracts was 1 mg/mL. The negative control was a solution with a concentration of methanol-water (80:20, v/v) containing the inoculum. A well with MHB plus inoculum was used as a positive control to discard any influence of methanol in the inhibitory activity of the crude extracts. A medium with no inoculum was applied to control sterility, and a well with a working solution (crude extract) without inoculum was used as a crude extract control. The inhibitory activity of the crude extracts was measured by the unaided eye by comparing the results obtained with the negative control. Inhibition was considered when a clear well was observed by the unaided eye.

Screening of the *P*. *zonale* fractions against *S*. *aureus* ATCC 25923 was performed as explained above with some modifications. A 96-well U-shaped plate (Tecan Group Ltd., Switzerland) was filled with 50 μL of working solution and allowed methanol evaporation for 30 min. The final reaction volume was 100 μL and the final concentration of the fractions was 800 μg/mL. The negative control was a solution with a concentration of methanol-water (20:80, v/v). The inhibitory activity of the crude extracts was measured by plating 20 μL of samples from clear wells onto NA plates without plant extract. The agar plates were incubated at 37°C for 24 h. Results are reported as colony-forming units per milliliter (CFU/mL).

### Drug susceptibility assays

Minimum inhibitory concentration (MIC) and minimal bactericidal concentration (MBC) of *P*. *graveolens*, *P*. *odoratissimum*, and *P*. *zonale* methanolic extracts were performed using the broth microdilution technique according to the Clinical and Laboratory Standards Institute (https://clsi.org/) [30] recommendations with some modifications. Bacterial suspensions were prepared as explained above. Working solutions of crude plant extracts were prepared by diluting the stock solutions with MHB to a final concentration of 4000 μg/mL. A 96-well U-shaped plate was filled in the first wells with 100 μL of working solution and allowed methanol evaporation for 30 min. Then, 100 μL of MHB was added to the first well, mixed, and 100 μL was taken from the first well to do 2-fold serial dilutions. After that, 100 μL of bacterial suspension (5 × 10^5^ CFU/mL) was added to each well. The microplate was incubated at 37°C between 16 to 20 h. The final concentrations of plant crude extracts were between 15.6 to 1000 μg/mL. The same controls as explained above were prepared with the addition of ciprofloxacin (Sigma-Aldrich, USA) used as a positive control with concentrations ranging from 0.09 to 3 μg/mL. The MIC was measured by the unaided eye by comparing the results obtained with the negative control. MBC was determined following the MIC assay, by plating 3 μL of samples from clear wells onto NA plates without plant extract. The agar plates were incubated at 37°C for 24 h. MBC was estimated as the least sample concentration where no visible microbial growth was observed.

### Time-kill assays

The methanolic extracts of *P*. *graveolens*, *P*. *odoratissimum*, and *P*. *zonale* were tested at concentrations corresponding to 4X MIC to evaluate their bactericidal or bacteriostatic effect according to the Clinical and Laboratory Standards Institute guidelines [31] with some modifications. The positive control ciprofloxacin was set at 4X MIC for the susceptible strains *E*. *faecalis* ATCC 29212 (3 μg/mL) and *S*. *aureus* ATCC 25923 (1.5 μg/mL). The negative control was a solution with a concentration of methanol-water (80:20, v/v). The experiment was performed in a 96-well round-bottom plate following the same conditions as explained above and incubated at 37°C for 24 h. At defined time intervals (4, 6, 8, 16, and 24 h), 20 μL of serial 10-fold dilutions were plated on NA (without plant extract) and incubated at 37°C for 24 h. Killing curves were constructed by plotting the Log_10_ CFU/mL versus time over 24 h. Bactericidal activity was defined as a decrease of more than 1000 times (3 Log_10_) of viable bacteria compared to the original inoculum.

### Biofilm inhibition and eradication assays

The biofilm inhibition and eradication activity of *P*. *graveolens*, *P*. *odoratissimum*, and *P*. *zonale* methanolic extracts were then evaluated as previously described [12]. Briefly, in both assays, an overnight culture growth was used to prepare a bacterial suspension with a final concentration of 5 × 10^7^ CFU/mL in MHB. In the inhibition assays, 190 μL of the inoculum was added to 96-well flat bottom plates and supplemented with 10 μL of crude extracts to achieve 1X and 2X MIC concentrations in each well. Plates were incubated for 24 h at 37°C. Regarding the eradication assays, the same bacterial suspension in MHB was prepared in 96-well plates incubating them for 48 h at 37°C. Subsequently, the MHB was removed from the wells, and a wash with PBS was performed. The mature biofilms were supplemented with crude extracts at 1X and 2X MIC concentrations in flesh media. Plates were incubated for 24 h at the same conditions. At the end of inhibition or eradication assays, three washes were performed in all plates with PBS, and each biofilm sample was fixed with 200 μL of methanol for 15 min. Crystal violet staining (0.1% w/v; Sigma-Aldrich, St. Louis, MO, USA) was performed for 10 min, followed by four washing steps with distilled water. After ethanol (95% v/v; Sigma-Aldrich, St. Louis, MO, USA) resuspension, each biofilm sample was measured at 570 nm in the ELISA Elx808 spectrophotometer (BioTek, Winooski, GU, USA) comparing with positive controls (bacteria growth in media only). Finally, the biofilm inhibition/eradication percentage was calculated as previously described [12]. A negative control was a solution of methanol-water (80:20, v/v), and a well with merely medium was applied as a sterility control. All the biological assays were set up in triplicates in at least two independent experiments.

### Cytotoxicity assays

MTT (3-[4,5-dimethylthiazol-2-yl]-2,5 diphenyl tetrazolium bromide) assay was performed as previously described [32]. Briefly, Raw 264.7 murine macrophages (ATCC) were seeded in 96-well flat-bottom culture plates (Corning, USA) at a density of 8 × 10^3^ in 200 μL of DMEM containing 10% FBS and cultured overnight. The following day media was removed and fresh DMEM was added supplemented with methanolic extracts at concentrations of 0, 125, 250, 500, and 1000 μg/mL. After 24 h, cells were washed once with Dulbecco’s phosphate buffered saline (PBS, pH 7.4; Sigma-Aldrich, St. Louis, MO, USA), and 200 μL of fresh DMEM was added. At 48 h, media was removed and changed for 200 μL of fresh DMEM with 1% FBS. Cell viability was assessed by adding 50 μL of filter sterilised MTT (5 mg/mL in PBS, Sigma-Aldrich, USA) to each well, followed by a 2 h incubation period at 37°C in 5% CO_2_. Media was removed and the blue formazan crystals trapped in cells were dissolved by adding 200 μL of DMSO and 25 μL of Sorensen’s glycine buffer. The absorbance at 570 nm was measured in a spectrophotometer. The percentage of viability was calculated relative to the control (DMEM media with the dissolvent methanol-water 80:20, v/v).

### Data processing and statistical analysis

MzMine V3.9.1 was used for data visualisation, mass detection, chromatogram builder, local minimum identification, MS1/MS2 building, isotope grouping, alignment, filtering, and gap filling [33] for the comparison of the *P*. *zonale* fractions. The resulting comma-separated values (cvs.) and mascot generic format (mgf.) files were used to compare differences between fractions. The metabolite area was used as an indicator of concentration. The metabolite areas in each fraction were obtained and then scaled using the Pareto method [34] with the following formula:

$${\tilde{x}}_{ij}=\frac{x_{ij}-{\bar{x}}_{i}}{\sqrt{s_{i}}}$$

Where: ${\bar{x}}_{i}=\frac{1}{J}\sum_{j=1}^{J}x_{ij}$ and $s_{i}=\sqrt{\frac{\sum_{j=1}^{J}{\left(x_{ij}-{\bar{x}}_{i}\right)}^{2}}{J-1}}$ to compare the different fractions.

Hierarchical clustering was done using Ward´s method as previously described [35]. For the comparison between chemical compounds, we used structural and physicochemical similarities computed with ChemMine Tools [36]. For pairwise comparison between control and treated samples in biofilm inhibition and eradication assays the Wilcoxon non-parametric test was used through R studio version 4.0 (https://www.rstudio.com/products/rstudio/download/) using several R packages ("ggpubr", "rstatixs", "openxlsx", and "tidyverse") [37]. For analysis of phytochemical screening and antioxidant activity, the IBM SPSS Statistics software for Windows version 25.0 (SPSS Inc., Chicago, IL, USA) was used and statistical analysis between different groups was performed using one-way ANOVA and Bonferroni post hoc tests. All *p*-values <0.05 were considered significant.

## Results and discussion

### Phytochemical profile and antioxidant activity of *P*. *odoratissimum*, *P*. *graveolens*, and *P*. *zonale*

The hydroalcoholic extracts of *P*. *odoratissimum*, *P*. *graveolens*, and *P*. *zonale* were analysed qualitatively and quantitatively for their phytochemical profile (Table 2). Overall, the total phenolic content (TPC) for the three *Pelargonium* species was higher than 200.0 mg GAE per g DE, with *P*. *zonale* showing the highest values of total phenolic compounds (350.2 mg GAE per g DE) and total tannin content (TTA) (142.3 TAEq per g DE) when compared to *P*. *odoratissimum* and *P*. *graveolens* (*p*-value < 0.05). *P*. *graveolens* extract showed higher TPC values (210.2 mg GAE per g DE) than those reported in previous studies when using methanolic extracts (145.0 mg GAE per g DE [38] and 84.2 mg GAE per g DE [39] and essential oils (102.4 mg GAE per g DE) [40]. In the case of *P*. *odoratissimum* extract, the TPC value correlates with our previous report [12] and is currently the only data available for comparison. The data available for *P*. *zonale* regarding TPC are not comparable with our methodology [9,17]. On the other hand, the total flavonoid content (TFC) of *P*. *graveolens* extract was the highest (49.2 mg EC per g DE) from our study set (Table 2), showing significant differences when compared to *P*. *zonale* and *P*. *odoratissimum* (*p*-value < 0.01).

**Table 2:** Phytochemical composition and total antioxidant capacity of *P*. *graveolens*, *P*. *odoratissimum*, and *P*. *zonale* hydroalcoholic extracts.

Parameter	TPC (mg GAE per g DE)	TFC (mg CE per g DE)	ACY (mg PgEq per g DE)	TTA (TAEq per g DE)	FAA (mg LEeq per g DE)	TAC (mmol TEq per g DE)	
						FRAP	DPPH
*P*. *graveolens*	210.0 ± 3.0 ^a^	49.2 ± 2.2 ^a^	ND	74.3 ± 4.8 ^a^	41.7 ± 6.3 ^a, b^	906.6 ± 50.5 ^a^	2476.7 ± 226.4 ^a^
P. *odoratissimum*	219.2 ± 8.0 ^a^	29.6 ± 1.1 ^b^	ND	82.3 ± 10.9 ^a^	43.2 ± 4.1 ^a^	937.1 ± 15.1 ^a^	2587.0 ± 141.6^a^
*P*. *zonale*	350.2 ± 9.1 ^b^	31.9 ± 0.5 ^b^	142.1 ± 8.5	142.3 ± 15.8 ^b^	29.9 ± 5.3 ^b^	756.0 ± 28.5 ^b^	3538.9 ± 145.7 ^b^
*p-*value	<0.01	<0.01		0.01	0.042	0.01	0.01

Results are expressed as mean ± standard deviation (SD). TPC: Total phenolic content, TFC: Total flavonoid content, ACY: Anthocyanin content, TTA: Total tannin content, FAA: Total free amino acid content, TAC: Total antioxidant capacity. FRAP: Ferric reduction antioxidant power, DPPH: 2,2-diphenyl-1-picrylhydrazyl free radical-scavenging ability., ND: Non-detected. Mean values within a column sharing the same letter are not significantly different after Bonferroni post-hoc analysis (*p*-value < 0.05).

Only *P*. *zonale* methanolic extract showed a detectable anthocyanin content (ACY) of 142.1 mg PgEq per g DE (Table 2), likely due to the presence of its characteristic red flowers in the extract preparation (Table 1), as others have previously reported [41]. No anthocyanin content was detected in the case of *P*. *graveolens* and *P*. *odoratissimum* methanolic extracts, probably due to the lack of flowers in the extract preparation (Table 1). Other studies have also shown the presence of anthocyanins in dried aerial parts (also containing flowers) and flower extracts of *P*. *graveolens* (0.28–1.01 g cyanine chlorine/100 g herbal product) [42,43] and in the pink-white flowers of *P*. *odoratissimum* (0.48–0.65 mg of malvin chloride/g fresh flowers) [44]. Thus, the anthocyanin content could be associated with the presence of flowers in the extract preparation. In the case of free amino acid content (FAA), the highest values were found in *P*. *odoratissimum* (43.2 mg LEeq per g DE), followed by *P*. *graveolens* and *P*. *zonale* (Table 2). No data on FAA is available on these three *Pelargonium* species being this study the first report.

The three methanolic extracts showed strong antioxidant activity as measured by DPPH (>1000 mmol TEq per g DE) (Table 2), with *P*. *zonale* showing the highest free radical-scavenging activity (3538.9 mmol TEq per g DE). In contrast, *P*. *zonale* had the lowest FRAP value (756.0 mmol TEq per g DE), but all three extracts showed medium FRAP values (756 to 937 mmol TEq per g DE). Significant differences (*p*-value < 0.01) were observed in both assays when comparing *P*. *zonale* with *P*. *graveolens* and *P*. *odoratissimum*.

Other authors have reported the antioxidant activity of *P*. *graveolens* [3] and *P*. *odoratissimum* [11,12] extracts. However, we cannot make comparisons due to differences in how the results were reported. To the best of our knowledge, there is no information available on the antioxidant activity of *P*. zonale. Overall, the three species have notable antioxidant activity regardless of the presence of flowers in the extract preparation. Considering that the total phenolic content (TPC) values in the three *Pelargonium* species were 4–10 times higher than the total flavonoid content (TFC), the antioxidant capacity of these methanolic extracts may have resulted from the high content of total phenolic compounds.

Phenolic antioxidants are highly efficient and typically required in very small amounts to neutralise many free radicals [45]. According to literature reports [45], the phenolic antioxidants identified in this study for *P*. *graveolens* include quercetin and kaempferol derivatives and a type of epi-gallocatechin and myricetin derivatives (Table 3). For *P*. zonale, quercetin and kaempferol derivatives, along with coumaroylquinic acid and anthocyanins derivatives, were identified. Lastly, for *P*. *odoratissimum*, quercetin and kaempferol derivatives and a type of epi-gallocatechin were identified.

**Table 3:** Tentative identification of the most abundant masses in *Pelargonium* species hydroalcoholic extracts.

ID	*P*. *g*	*P*. *o*	*P*. *z*	Parent mass *m/z*	Main MS/MS products (*m/z*)	Tentative identification	References
	Rt (min)						
**1**			1.15	381 [M+K]^+^	201(100) 219(90) 249(25) 363(25) 207(25) 235(25)	Sucrose	[46]
**2**			1.53	308 [M+H]^+^	179(100) 162(25) 233(15) 290(5)	L-Glutathione	89.8%
**3**			2.35	322 [M+H]^+^	193(100) 176(20) 233(10) 304(5)	L-l-Homoglutathione	[47]
**4**	4.98	4.90		611 [M+H]^+^	443(100), 317(30) 425(20) 307(10) 287(5)	A type of (epi)gallocatechin dimer	[48,49]
**5**			5.58	678 [M+H]+	354(100) 516(80)	UI	
**6**		6.23		220 [M+H]^+^	202(100) 90(40) 184(30)	Pantothenic acid	90.0%
**7**			6.68	205 [M+H]^+^	188(100)	DL-Tryptophan	91.9%
**8**	5.61	6.81		307 [M+H]^+^	139(100) 289(95) 150(80) 181(10)	(-)-Epigallocatechin	92.7%
**9**	6.96	7.41		205 [M+H]^+^	188(100), 159 (5)	DL-Tryptophan	90.9%
**10**	8.45			1219 [M+H]^+^	915(100) 1051(30) 895(30) 1093(10) 1134(10) 757(5) 699(5) 611(5)	A type of (epi)gallocatechin tetramer	[48]
**11**	8.55			305 [M+H]^+^	287(100) 127(70) 143(60) 277(50) 179(20)	UI	See comments
**12**	8.20	9.86		611 [M+H]^+^	425(100) 287(87) 443(82) 317(61) 307(55) 305(34)	A type of (epi)gallocatechin dimer	[48]
**13**	9.11	9.67		915 [M+H]^+^	609(100) 611(75) 747(25) 836(20)	A type of (epi)gallocatechin trimer	[48]
**14**			9.44	395 [M+H]^+^	233(100) 377(35) 246(25) 215(10) 132(10)	UI	UI
**15**	9.46			1523 [M+H]^+^	1183(100) 895(95) 1216(80) 851(70) 1016(60)	A type of (epi)gallocatechin pentamer	[49]
**16**		9.61		915 [M+H]	747(100) 609(80) 739(40) 878 (25) 611(35)	A type of (epi)gallocatechin trimer	[48]
**17**			9.66	611 [M+H]^+^	287(100) 449(75)	Cyanidin 3,5-diglucoside	[50]
**18**		9.81		295 [M+H]^+^	166(100) 120(20)	Gamma-glutamylphenylalanine	MassBank Record: MSBNK-Metabolon-MT000057
**19**	10.18			291 [M+H]^+^	123(100) 139(80) 273(60) 165(40) 245(30)	Catechin	80.8%
**20**			10.3	595 [M]^+^	433(100) 271(98) 576(14) 325(5)	Pelargonidin 3,5-diglucoside	[50]
**21**	10.34			344 [M+H]^+^	165(100) 147(50) 309(20) 180(10)	4-*O*-coumaroyl–amino glycoside	[51]
**22**			10.65	625 [M]^+^	463(100) 301(80) 286(5)	Peonin	[47]
**23**	10.98	11.17		389 [M+H]^+^	209(100) 227(30) 371(10) 191(10)	4-[4-hydroxy-2,6,6-trimethyl-3-[3,4,5-trihydroxy-6-(hydroxymethyl)oxan-2-yl]oxycyclohexen-1-yl]butan-2-one	82.2%
**24**			11.93	339 [M+H]^+^	147(100) 321(5) 119(5)	Coumaroyl quinic acid	78.1%
**25**	12.18			393 [M+H]^+^	197(100) 375(60) 285(40) 345(20)	UI	
**26**			12.18	433 [M+H]^+^	271(100)	Pelargonidin galactoside	[52]
**27**			12.39	611 [M+H]^+^	449(100) 287(25)	Luteolin 3’,4’-*O*-beta-D-glucopyranoside	81.0%
**28**	12.91			613 [M+H]^+^	319(100) 481(50) 595(20)	Myricetin 3-sambubioside	See comments
**29**		12.91		502 [M+H]^+^	373(100) 355(50) 209(10) 484(10)	N5-((S)-1-((carboxymethyl)amino)-3-(((R)-1-(3,4-dimethoxyphenyl)-3-hydroxypropan-2-yl)thio)-1-oxopropan-2-yl)-L-glutamine	[53]
**30**	13.09			369 [M+H]^+^	193(100) 235(50) 351(50) 333(40) 176(25) 215(25) 277(20)	UI	
**31**			13.24	595 [M+H]^+^	449(100) 287(30) 576(20) 433(20)	UI	
**32**	13.3		13.29	627 [M+H]^+^	303(100) 465(80) 319(30) 609(10)	Quercetin 3,4’-diglucoside	[54]
**33**	13.48			481 [M+H]^+^	319(100) 462(5)	Myricetin 3-*O*-beta-D-galactopiranose	90.3%
**34**	13.83			597 [M+H]^+^	303(100) 465(50) 435(10)	Quercetin-3-*O*-vicianoside	79.4%
**35**	14.39			451 [M+H]^+^	319(100) 329(5)	Myricetin pentoside	See comments
**36**	14.47	14.52	14.42	611 [M+H]^+^	303(100) 465(30)	Rutin	89.8%
**37**	14.68		14.67	465 [M+H]^+^	303(100)	Quercetin hexoside	See comments
**38**			14.78	595 [M+H]^+^	287(100) 449(40) 433(10) 576(5)	Kaempferol glucorhamnoside	91.0%
**39**		14.96		433 [M+H]^+^	415(100), 367(50), 271(40), 397(25), 337(20), 313(20), 379(15)	Vitexin	87.3%
**40**	15.36		15.95	595 [M+H]^+^	287(100) 449(30) 576(5)	Kaempferol glucorahamnoside (isomer 1)	97.6%
**41**	15.63		15.47	435 [M+H]^+^	303(100)	Quercetin pentoside	See comments
**42**		15.71		437 [M+H]^+^	419(100) 341(30) 383(25) 317(25) 401(15)	p-Phlorizin	87.1%
**43**	15.98			373 [M+H]^+^	211(100) 193(20) 355(20) 175(10) 135(10)	4-hydroxy-5-methoxy-trans-melilotoside	[55]
**44**	16.02			625 [M+H]^+^	317(100) 479(25) 593(5)	Rhamnetin-3-rutinoside	78.1%
**45**	16.17	16.26		449 [M+H]^+^	287(100) 430(5)	Trifolin	89.9%
**46**	16.58		16.38	419 [M+H]^+^	287(100) 401(5)	Juglanin	87.0%
**47**	17.12			275 [M+H]^+^	107(100) 149(50) 169(30) 127(20)	Phloretin	87.1%
**48**	17.30			679 [M+H]^+^	661(100) 647(80) 623(30) 522(10)	Ov-NCC-1	[56]
**49**			18.09	491 [M+H]^+^	303(100) 189(90) 473(60) 459(20)	Quercetin 3-*O*-acetyl-rhamnoside	FooDB database: compound FDB000163

Unidentified compound (UI). The percentage indicates the score reported in the MzCloud database. *P*.*g*: *P*. *graveolens*, *P*.*o*: *P*. *odoratissimum* and *P*.*z*: *P*. *zonale*. FooDB accessible at: https://foodb.ca/.

### Chemical characterisation of *P*. *graveolens*, *P*. *odoratissimum*, and *P*. *zonale*

The chemical characterization of the most abundant compounds from the three *Pelargonium* species was analysed by high-performance liquid chromatography-diode array detector-tandem mass spectrometry (HPLC-DAD-MS/MS). The tentative identification of the parent ions in positive ion mode is shown in Table 3 and a detailed discussion about these identifications is presented in S1 Table.

Several bioactive compounds were identified in the three *Pelargonium* species, mainly phenolic acids, flavonoids, tannins, and chalcones. A previous study also described the presence of myricetin, quercetin, and kaempferol flavonoids, or their derivatives, in several *Pelargonium* species [57]. The flavonoids identified in *P*. *graveolens* are consistent with those reported by Boukhris et al. (2013) [39]. Moreover, among the three species studied, *P*. *graveolens* stands out due to the various types of myricetin derivatives that were not detected in *P*. *odoratissimum* and *P*. *zonale*. On the other hand, *P*. *odoratissimum* showed a chemical composition similar to the one reported in our previous work [12], comprising flavonoids, gallocatechin and epigallocatechin tannins, and gallic acid and derivatives. Gallocatechin-derived tannins, which are known for their antimicrobial activity [58], were detected in *P*. *odoratissimum* and *P*. *zonale*. It should be noted that no in-depth study has been conducted on the chemical characterisation of *P*. *zonale* hydroalcoholic extracts, and the existing information is based only on the results of HPLC analyses [9,17]. In addition to the reported compounds in *P*. *zonale*, four anthocyanins were identified in the present work, more exactly cyanidin 3,5-diglucoside, pelargonidin-galactoside, pelargonidin 3,5-diglucoside, and peonin. The latter two compounds are the most described constituents in the *Pelargonium* genus [59] and are particularly reported in species with red flowers [60].

### Screening of the antimicrobial activity of *P*. *graveolens*, *P*. *odoratissimum*, and *P*. *zonale*

Next, we investigated the antimicrobial activity of *P*. *graveolens*, *P*. *odoratissimum*, and *P*. *zonale* methanolic extracts using a microdilution broth assay against a set of seven susceptible bacterial strains and two susceptible yeast strains (Table 4). These strains were chosen as representatives of some of the most common microbial pathogens that are critical to human health. The initial screening was carried out at a maximum single-point concentration of 1000 μg/mL. Overall, these three methanolic extracts showed visible growth inhibition only towards Gram-positive bacteria (Table 4). We did not observe antimicrobial activity from any of the three *Pelargonium* species towards the Gram-negative bacteria tested nor for *Candida* species at the maximum concentration of 1000 μg/mL.

**Table 4:** Screening of the antimicrobial activity of *P*. *graveolens*, *P*. *odoratissimum*, and *P*. *zonale* methanolic extracts against bacterial and yeast strains of clinical importance using a microdilution assay. Methanolic extracts are at a concentration of 1000 μg/mL.

N	Plant scientific name	Bacterial Strains									Yeast Strains	
		*E*. *faecium* ATCC 27270	*E*. *faecalis* ATCC 29212	*E*. *faecalis* INSPI 032	*S*. *aureus* ATCC 25923	MRSA 333	*A*. *baumannii* ATCC 19606	*P*. *aeruginosa* ATCC 27853	*E*. *cloacae* ATCC 23355	*E*. *coli* ATCC 25922	*C*. *albicans* ATCC 10231	*C*. *tropicalis* ATCC 1369
1	*Pelargonium graveolens*	-	**+**	**+**	**+**	-	-	-	-	-	-	-
2	*Pelargonium odoratissimum*	-	-	NT	**+**	-	-	-	-	-	-	-
3	*Pelargonium zonale*	-	**+**	**+**	**+**	**+**	-	-	-	-	-	-
	Negative control	-	-	-	-	-	-	-	-	-	-	-

Visible complete growth inhibition is registered as (+) and the lack of inhibitory activity as (-). NT: Not tested. Negative control is methanol-water (80:20, v/v).

However, other authors reported antimicrobial activity against the Gram-negative bacteria *Klebsiella pneumoniae* (MIC = 312 μg/mL) [7] and *E*. *coli* [5] when using dried leaves of *P*. *graveolens* dissolved in methanol and ethanol, respectively. In addition, Lewtak and colleagues reported the antifungal activity of the flower stalks of *P*. *zonale* hydroalcoholic extract against *C*. *albicans* (some activity observed at 37.5 μg/mL) [8]. In our study, we used leaves and stalks to prepare *P*. *graveolens* extract and flowers, stalks, and leaves were used to prepare *P*. *zonale* extract, both dissolved with methanol. Therefore, the differences observed in the antimicrobial activity may be attributed to the alteration in quantity and quality of secondary products of plants by different factors such as changes in cultivation, origin of the plant, growing seasons, time of collection, temperature, soil water, light, part of the plant used for the extraction or infusion preparations, among others [61]. In addition, it is well-known that the differences in cell membrane structure and composition between Gram-negative and Gram-positive bacteria play a critical role in the susceptibility to plant phenolics [62].

To this point, we also evaluated if the antibacterial activity observed against the susceptible strains *E*. *faecalis* ATCC 29212 and *S*. *aureus* ATCC 25923 was also detected in the Vancomycin-resistant *E*. *faecalis* INSPI 032 and the Methicillin-resistant *S*. *aureus* (MRSA) 333 clinical strains (Table 4). Overall, *P*. *graveolens* methanolic extract showed complete visible growth inhibition against *E*. *faecalis* ATCC 29212, *S*. *aureus* ATCC 25923, and the Vancomycin-resistant *E*. *faecalis* INSPI 032 strains, while *P*. *zonale* inhibited the growth of all these three strains plus the MRSA 333 strain. In contrast, *P*. *odoratissimum* only showed complete growth inhibition against *S*. *aureus* ATCC 25923, in agreement with our previous report [12].

### Drug susceptibility assays

Confirmation of the hits from the initial screening was performed by calculating the minimum inhibitory concentration (MIC) (Table 5). The main difference between the MIC values of these three crude extracts was observed in the MIC of *P*. *zonale* towards *S*. *aureus* ATCC 25923, which was four times lower (MIC = 250 μg/mL) than the MIC values shown by *P*. *graveolens* and *P*. *odoratissimum* (MIC = 1000 μg/mL). In addition, among the three *Pelargonium* species, only *P*. *zonale* inhibited the growth of the MRSA 333 strain (MIC = 250 μg/mL).

**Table 5:** Growing inhibitory parameters (MIC, and MBC) of *P*. *graveolens*, *P*. *odoratissimum* and *P*. *zonale* methanolic extracts against bacteria of clinical importance using broth dilution format.

N	Plant scientific name	MIC				MBC			
		*E*. *faecalis* ATCC29212	*E*. *faecalis* INSPI 032	*S*. *aureus* ATCC25923	MRSA 333	*E*. *faecalis* ATCC29212	*E*. *faecalis* NSPI 032	*S*. *aureus* ATCC25923	MRSA 333
1	*Pelargonium graveolens*	1000	1000	1000	NT	2000	2000	2500	NT
2	*Pelargonium odoratissimum*	NT	NT	1000	NT	NT	NT	2500	NT
3	*Pelargonium zonale*	1000	1000	250	250	1500	1500	500	1000
	Ciprofloxacin	1.5	NT	0.38	NT	-	-	-	-

The MICs, and MBCs are represented in μg/mL. MIC: Minimum inhibitory concentration. MBC: Minimal bactericidal concentration. NT: Not tested. Ciprofloxacin was used as a positive control for susceptible bacterial strains.

Hsouna and Hamdi reported a MIC value of 2500 μg/mL against *S*. *aureus* ATCC 25923 using leaves of *P*. *graveolens* to prepare a methanolic extract, but no activity towards *E*. *faecalis* ATCC 29212 was observed [7]. This is in contrast with our results displaying MIC values of 1000 μg/mL for both strains. In the case of *P*. *zonale* and *P*. *odoratissimum*, studies on antimicrobial activities are scarce and mainly reported using essential oils [10,13,14], ethanolic extracts [8] or in combination with zinc oxide (ZnO) nanoparticles using *P*. *odoratissimum* aqueous leaf extract [11]. It should be noted that the antibacterial activity of *P*. *odoratissimum* methanolic extracts has only been previously reported by us [12], showing MIC values against *S*. *aureus* ATCC 25923 similar to this current report.

Overall, *P*. *zonale*’s antibacterial activity against Gram-positive bacteria was shown to be superior to the activity presented by *P*. *graveolens* and *P*. *odoratissimum* (Table 5). This greater activity may be related to the differences in the quantity of the phenolic content, especially of anthocyanins, which are only present in *P*. *zonale* (Table 2). For instance, anthocyanins such as cyanidin and pelargonidin detected in pomegranate fruit were found to be active against a broad range of Gram-positive bacteria, including *S*. *aureus* and *E*. *faecalis* [63]. However, Cisowska and colleagues have pointed out that the antimicrobial activity of anthocyanins is likely to be caused by multiple mechanisms and synergies involving a variety of different chemical compounds [64].

Likewise, there are reports of polyphenolic extracts with antibacterial activity against *S*. *aureus* and MRSA strains where the inhibitory activity was attributed to the presence of compounds such as myricetin, quercetin and kaempferol derivatives, (-)-epi-gallocatechin, and coumaroylquinic acid, among others [65]. The same type of compounds were also identified in this study.

### Bactericidal activity of *P*. *graveolens*, *P*. *odoratissimum*, and *P*. *zonale*

Next, to assess if *P*. *graveolens*, *P*. *odoratissimum*, and *P*. *zonale* methanolic extracts exhibit bactericidal or bacteriostatic activity, we determined the minimal bactericidal concentration (MBC) (Table 5). The crude extracts showed an MBC/MIC ratio value lower or equal to four, indicating bactericidal activity [31]. Then, to further validate the effects of the methanolic extracts on the growth kinetics of *E*. *faecalis* ATCC 29212, *S*. *aureus* ATCC 25923, the Vancomycin-resistant *E*. *faecalis* INSPI 032, and MRSA 333 strains, time-kill assays were performed (S1 Fig).

Bactericidal activity is defined as 99.9% killing and can be calculated from time-kill curves when reaching a decrease in bacterial growth ≥ 3 Log_10_ [31]. Overall, *P*. *graveolens*, *P*. *odoratissimum*, and *P*. *zonale* at 4X MIC showed a decrease of 3 Log_10_ between 6 to 8 h, while the positive control ciprofloxacin at 4X MIC exhibited bactericidal activity at 4 h. Thus, the time-kill values support the MBC data and showed that *P*. *graveolens*, *P*. *odoratissimum*, and *P*. *zonale* exhibited bactericidal activity against *E*. *faecalis* and *S*. *aureus* susceptible and resistant strains.

### Biofilm inhibition and eradication assays

**Fig 1:**
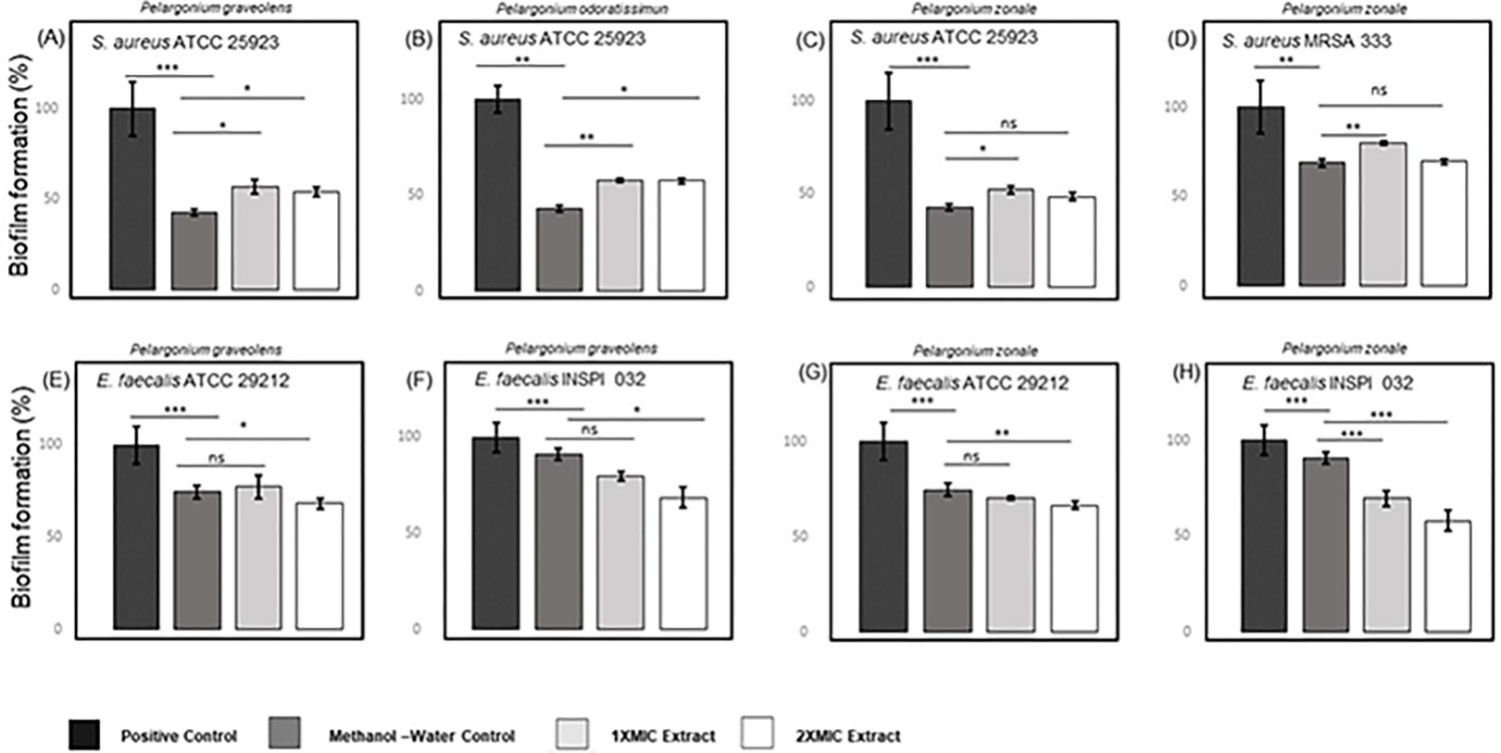
Representative illustration of the biofilm inhibition by *P*. *graveolens*, *P*. *odoratissimum* and *P*. *zonale* against bacteria of clinical importance. The plots illustrated the biofilm inhibition of *S*. *aureus* ATCC 25923 by (A) *P*. *graveolens*, (B) *P*. *odoratissimum*, and (C) *P*. *zonale*. Biofilm inhibition of *S*. *aureus* MRSA 333 by (D) *P*. *zonale*, followed by the biofilm inhibition of (E) *E*. *faecalis* ATCC 29212 and (F) *E*. *faecalis* INSPI 032 by *P*. *graveolens*. Finally, biofilm inhibition of (G) *E*. *faecalis* ATCC 29212 and *E*. *faecalis* INSPI 032 (H) by *P*. *zonale*. Biofilm formation values of methanol-water controls and samples were calculated as the percentage of bacteria biofilm formation through the optical density comparison between methanol-water controls/samples and bacterial growth in only medium culture (positive control). Error bars indicate ± SD of two independent experiments. Statistical significance was analysed using a non-parametric Wilcoxon test (95% confidence interval) for comparison between biofilm formation values where: * *p*-values < 0.05; ** *p*-values < 0.01; *** *p*-values < 0.001; and ns: Non-significant *p*-value.

**Fig 2:**
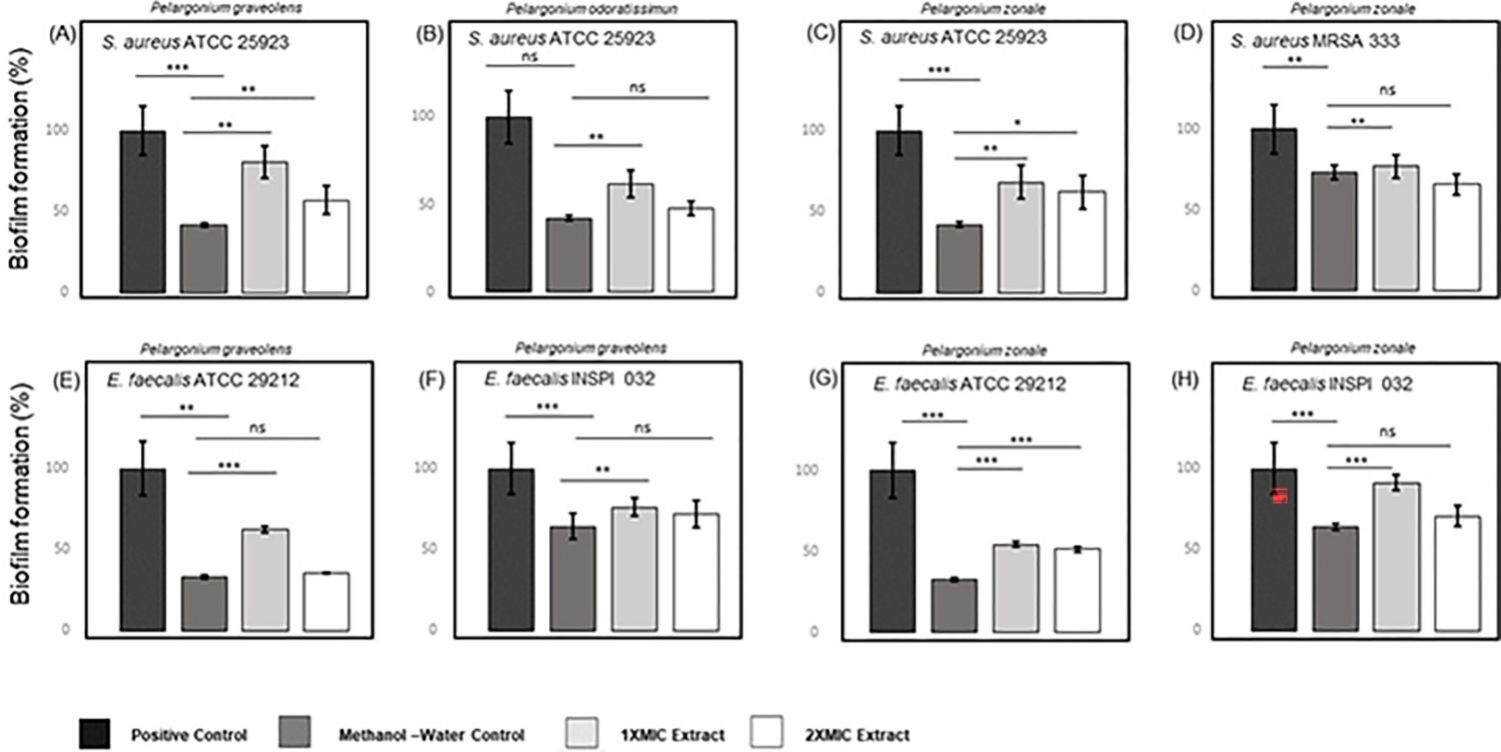
Representative illustration of the biofilm eradication by *P*. *graveolens*, *P*. *odoratissimum* and *P*. *zonale* against bacteria of clinical importance. The plots illustrated the biofilm eradication of *S*. *aureus* ATCC 25923 by (A) *P*. *graveolens*, (B) *P*. *odoratissimum*, and (C) *P*. *zonale*. Biofilm eradication of *S*. *aureus* MRSA 333 by (D) *P*. *zonale*, followed by the biofilm eradication of (E) *E*. *faecalis* ATCC 29212 and (F) *E*. *faecalis* INSPI 032 by *P*. *graveolens*. Finally, biofilm eradication of (G) *E*. *faecalis* ATCC 29212 and *E*. *faecalis* INSPI 032 (H) by *P*. *zonale*. Biofilm formation values of methanol-water controls and samples were calculated as the percentage of bacteria biofilm formation through the optical density comparison between methanol-water controls/samples and bacterial growth in only medium culture (positive control). Error bars indicate ± SD of two independent experiments. Statistical significance was analysed using a non-parametric Wilcoxon test (95% confidence interval) for comparison between biofilm formation values where: * *p*-values < 0.05; ** *p*-values < 0.01; *** *p*-values < 0.001; and ns: Non-significant *p*-value.

The inhibition and eradication effect of *P*. *graveolens*, *P*. *odoratissimum*, and *P*. *zonale* extracts was also studied against biofilms of *S*. *aureus* ATCC 25923, MRSA 333, *E*. *faecalis* ATCC 29212, and the Vancomycin-Resistant *E*. *faecalis* INSPI 032 (Figs 1 and 2, S2 Table). *P*. *graveolens*, *P*. *odoratissimum*, and *P*. *zonale* were applied at 1X and 2X MIC concentrations against the evaluated bacterial pathogens (Figs 1 and 2). When compared to the positive control (bacteria and media only), the range of the overall inhibition percentage values of the extracts against the evaluated bacterial pathogens was between 20.3 and 51.3% (Fig 1), while the eradication values were between 8.7 and 64.8% (Fig 2). All the negative methanol-water controls (80:20, v/v) showed statistical differences when compared with the positive control, indicating a biofilm inhibition or eradication by itself. It is important to mention that the methanol evaporation step performed in all previous drug susceptibility assays was not done as it interferes with the procedure in both biofilm inhibition and eradication assays. The lack of methanol evaporation clearly showed a greater inhibitory/eradication activity of the negative controls when compared with the positive control and overestimated biofilm inhibition and eradication effects on the evaluation of the plant extracts. Hence, when counteracted with the methanol-water control, no inhibition effects were observed in *S*. *aureus* ATCC 25923 and MRSA 333 biofilms when using *P*. *graveolens*, *P*. *odoratissimum*, and *P*. *zonale* extracts (Fig 1A–1D).

On the other hand, *P*. *graveolens* and *P*. *zonale* extracts evidenced significant biofilm inhibition values against *E*. *faecalis* ATCC 29212 and the Vancomycin-resistant *E*. *faecalis* INSPI 032 strains (*p*-values < 0.05), independently of the presence of methanol, demonstrating higher inhibition values at 2X MIC concentration (Fig 1E–1H). More specifically, at 2X MIC (2000 μg/mL), *P*. *graveolens* and *P*. *zonale* slightly inhibited biofilm formation by 7.9 and 6.3% against *E*. *faecalis* ATCC 29212 (Fig 1E and 1G), respectively, while the Vancomycin-Resistant *E*. *faecalis* INSPI 032 showed 32.9 and 22.5% biofilm inhibition when counteracted by the methanol-water control (Fig 1F and 1H). Reports of *P*. *graveolens* antibiofilm activity have been found using ethanolic extracts and essential oils. For instance, *P*. *graveolens* ethanolic extract showed antibiofilm activity against *Streptococcus pyogenes* ATCC 19615, ATCC 49399, and one clinical isolate with MIC and MBC values superior to 1000 μg/mL [66]. To the best of the authors’ knowledge, no antibiofilm activity by *P*. *zonale* was previously reported.

Concerning the four anthocyanins (cyanidin 3,5-diglucoside, pelargonidin-galactoside, pelargonidin 3,5-diglucoside, and peonin) identified in *P*. *zonale* in this study, Jeyaraj and colleagues demonstrated that the aqueous anthocyanin-rich fraction of *Clitoria ternatea* flowers of Asia was responsible for the biofilm-inhibiting activity (around 64.0%) and also significantly reduced bacterial attachment of *Pseudomonas aeruginosa* ATCC 9027 [67]. However, the study did not identify individual anthocyanins from the anthocyanin-rich fraction that could be the active molecules responsible for the observed antibiofilm activity. Meanwhile, when compared to the present work, Zhang and colleagues identified similar anthocyanins (cyanidin, pelargonidin, and peonidin) in purple highland barley bran (PHBB) from the Tibetan Plateau in China, evidencing their antibiofilm activity against *P*. *aeruginosa* PAO1 and *Salmonella enterica* ATCC 10398 [68]. Furthermore, Pejin and colleagues evaluated the individual contribution of three anthocyanidins (pelargonidin, cyanidin, and delphinidin), demonstrating their antibiofilm effects on *P*. *aeruginosa* PAO1 [69]. Therefore, anthocyanins seem to contribute to the antibiofilm activity of *P*. *zonale* in the present study, but further studies are needed to analyse their individual antibiofilm potential and future applications.

Regarding the biofilm eradication assays (Fig 2), the plant extracts showed no eradication ability against *S*. *aureus* ATCC 25923, *E*. *faecalis* ATCC 29212 and the Vancomycin-resistant *E*. *faecalis* INSPI 032 biofilms except for MRSA 333 biofilms, which suffered a slight eradication of 7.2% by *P*. *zonale* at 2X MIC (500 μg/mL) when counteracted with the methanol-water control (Fig 2D). However, no statistical difference was observed. Overall, both *P*. *graveolens* and *P*. *zonale* extracts in the present study revealed biofilm inhibition against the Vancomycin-resistant *E*. *faecalis* INSPI 032 despite the presence of methanol, with *P*. *zonale* showing a slight biofilm eradication activity towards the MRSA 333 strain.

### Influence of *P*. *graveolens*, *P*. *odoratissimum*, and *P*. *zonale* on cell viability

**Fig 3:**
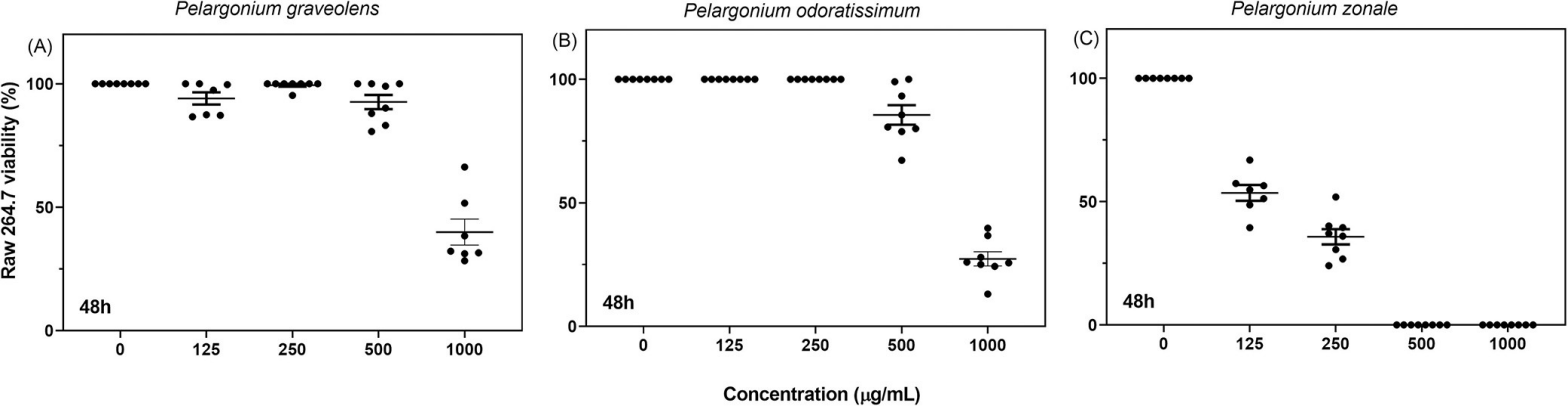
Raw 264.7 macrophage viability at 48 h upon treatment with the methanolic extracts. (A) *P*. *graveolens*, (B) *P*. *odoratissimum* and (C) *P*. *zonale* at concentrations of 0, 125, 250, 500 and 1000 μg/mL. The percentage of viability was calculated relative to the control (DMEM media with the dissolvent methanol-water 80:20, v/v). Error bars indicate ± SD of two independent experiments done in quadruplicates.

Next, we set to investigate the cytotoxicity effect of the three *Pelargonium* species in a dose-dependent manner (Fig 3). Macrophage viability with *P*. *graveolens* and *P*. *odoratissimum* at a concentration of up to 500 μg/mL was between 85 and 100%. A study evaluating *P*. *graveolens* acetone extract showed non-toxic effects at a concentration of 83 μg/mL [70]. However, *P*. *graveolens* and *P*. *odoratissimum* methanolic extracts at a concentration of 1000 μg/mL were toxic, with macrophage viability between 30 and 40% (Fig 3A and 3B). In contrast, *P*. *zonale* showed high cytotoxicity (macrophage viability of 50%) at the lowest concentration tested of 125 μg/mL, while no macrophage viability was observed at concentrations higher than 500 μg/mL (Fig 3C). This overall cytotoxicity effect might be related to the presence of tannins in *P*. *graveolens* and *P*. *odoratissimum*, as well as tannins and flavonoids (like anthocyanins) in *P*. *zonale* (Table 2). Tannins and flavonoids are reported as potential anticancer agents for their high levels of cytotoxicity in tumour cells [71]. Thus, the high cytotoxicity of *P*. *zonale* suggests that its antibacterial activity might be ascribed to its general cytotoxic effect. Indeed, *P*. *zonale* showed the best antibacterial activity among *P*. *graveolens* and *P*. *odoratissimum* methanolic extracts (Table 5). To the best of our knowledge, no data about the cytotoxicity of *P*. *odoratissimum* and *P*. *zonale* has been reported.

### Antibacterial activity of *P*. *zonale* fractions

**Fig 4:**
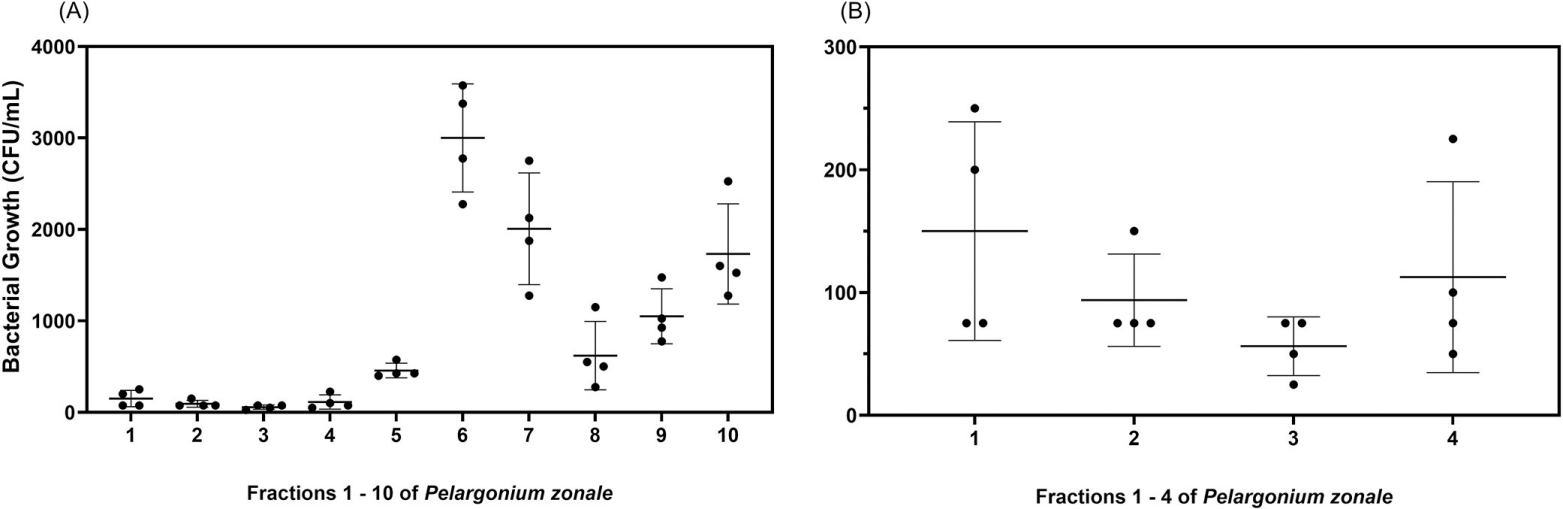
Antibacterial effect of *P*. *zonale* fractions in *S*. *aureus* ATCC 25923 growth. Fractions 1 to 10 (A) and fractions 1 to 4 (B) were evaluated against *S*. *aureus* ATCC 25923 at 800 μg/mL. Growth inhibition of the 14 fractions is expressed as CFU/mL. Error bars indicate ± SD of two independent experiments done in duplicates.

The best antibacterial activity was encountered in *P*. *zonale* methanolic extract when compared to *P*. *graveolens* and *P*. *odoratissimum* (Table 5), leading us to identify the active fractions. For this, *P*. *zonale* methanolic extract was fractionated with seven eluents using an SPE column (see methods section for details) to determine the tentative compounds exhibiting antimicrobial activity. A total of 14 fractions were obtained, from which fractions 1 to 10 were tested against *S*. *aureus* ATCC 25923 at a maximum single-point concentration of 800 μg/mL. The other fractions, 11 to 14, were not evaluated due to low solubility in methanol-water (20:80, v/v). *S*. *aureus* ATCC 25923 was selected as a representative of the Gram-positive bacteria tested in this study. Among all the fractions tested, fractions 1 to 4 showed the best antibacterial activity with colony-forming units (CFU) counts of less than 200 CFU/mL when compared with the other fractions (Fig 4A). Fraction 3 showed a slightly better antimicrobial activity (56 CFU/mL, Fig 4B) compared with fractions 1, 2, and 4 (150, 93, and 112. CFU/mL, respectively). Thus, the four fractions were further analysed by HPLC-DAD-MS/MS in positive mode.

### Chemical characterisation of *P*. *zonale* fractions

**Fig 5:**
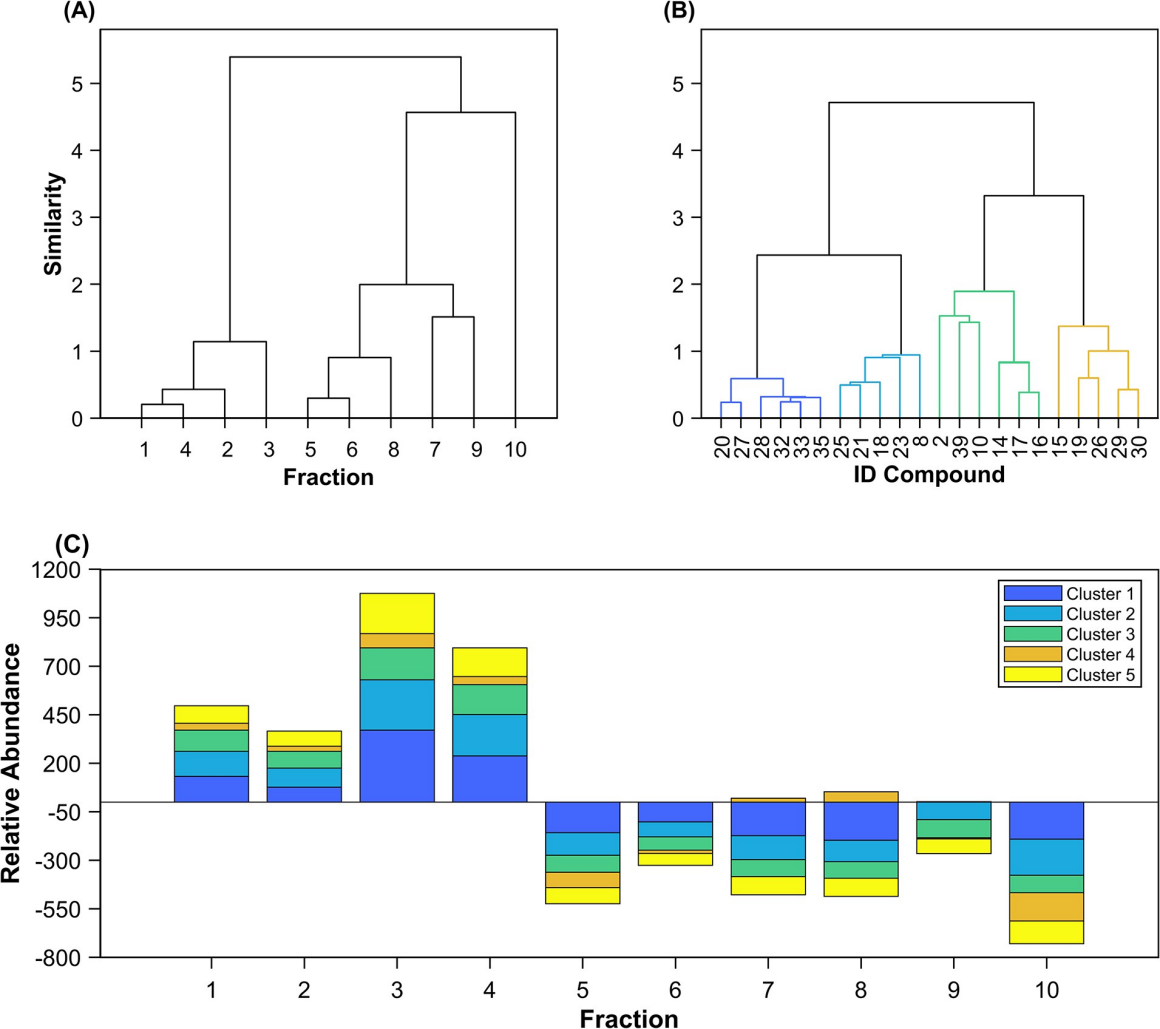
Overall evaluation of the chemical profile among fractions. (A) Dendrogram computed from fractions 1 to 10 post solid-phase extraction of *P*. *zonale* based on rescaled metabolite areas. Two clusters are observed comprising fractions 1–4 (cluster 1) and comprising fractions 5–10 (cluster 2). (B) Dendrogram based on tentatively identified compounds from fractions 1 to 4. Distances were computed from their structural and physicochemical similarities using ChemMine Tools. Four clusters are observed and are represented in blue (cluster 1), light blue (cluster 2), green (cluster 3) and orange (cluster 4). (C) The relative abundance of each chemical cluster in the fractions was determined by calculating the mean of the normalised areas of all compounds in the cluster. It should be noted that Cluster 5 (yellow), which corresponds to unidentified compounds, has been included.

A chromatographic analysis of fractions 1 to 4 (S2A Fig) revealed a similar chemical profile among them, which was also confirmed by a dendrogram (Fig 5A). The dendrogram is based on the rescaled areas of the metabolites present in a fraction, where areas are used as an indicator of metabolites’ relative concentration. The dendrogram’s elements represent individual fractions, with the distance between them reflecting similarities or differences in metabolite profiles. In Fig 5A, we can see two distinct clusters. The first cluster comprises fractions 1 to 4, and the second cluster comprises fractions 5 to 10. This clustering pattern may indicate a variation in the chemical composition of the analysed fractions, more exactly beyond the fourth fraction.

Considering the antibacterial activity (Fig 4), fractions 1 to 4 were more relevant and, consequently, the focus of further analysis. Several peaks were found in these fractions, and their tentative identifications are presented in Table 6. The chromatograms and a complete discussion about the tentative identification of compounds are shown in S2B Fig and S3 Table. Several compounds listed in Table 6 were already presented in Table 3 for *P*. *zonale*.

**Table 6:** Tentative identification of metabolites identified in fractions 1–4 in *P*. *zonale* in positive ionization mode.

ID	RT (min)	Parent mass *m/z*	Main MS/MS products (*m/z*)	Tentative identification	Resource
**1**	4.37	192 [M+H]^+^	122(100) 120(95) 82(74) 121(48) 123(32) 174(19) 183(17) 160(15) 83(11) 130(9)175(8) 70(6) 84(6) 161(6) 146(7)	UI	
**2**	4.89	381 [M+H]^+^	201(80) 213(5) 219(100)	Sucrose	[46]
**3**	4.91	147	147(100)	UI	
**4**	5.14	116	116(100)	UI	
**5**	5.26	258	104(100) 240(8)	UI	
**6**	5.77	678	336(16) 354(100) 498(10) 516(79)	UI	
**7**	6.00	132		UI	
**8**	6.07	595 [M]+	271(95) 433(100)	Pelargonidin 3,5-diglucoside	
**9**	6.11	166	166(100)	UI	
**10**	6.23	205 [M+H]^+^	188(100) 159(1)4	DL-Tryptophan	91.4%
**11**	6.69	487	143(8) 144(86) 161(100) 327(71) 469(6)	UI	
**12**	7.08	227	85(8) 167(5) 191(42) 209(100)	UI	
**13**	7.98	185	153(100)	UI	
**14**	8.28	339 [M+H]^+^	119(8) 147(100) 321(11)	Coumaroylquinic acid isomer 1	80.3%
**15**	8.64	619 [M-OH]^+^	233(5) 237(7) 261(12) 279(14) 297(6) 305(12) 431(6) 449(100) 467(14)	1,2,6-Trigalloylglucose	95.0%
**16**	8.98	369 [M+H]^+^	145(10) 177(100)	4-*O*-feruloyl-D-quinic acid	84.6%
**17**	9.16	339 [M+H]^+^	119(8) 147(100) 290(5) 321(11)	Coumaroylquinic acid isomer 2	80.8%
**18**	9.75	611 [M+H]^+^	303(100) 449(6) 465(28)	Rutin	96.1%
**19**	10.02	771 [M-OH]^+^	233(16) 243(9) 261(56) 279(100) 305(72) 413(12) 431(34) 449(21) 583(6) 601(13) 619(13)	Tetragalloylglucopyranose	74.7%
**20**	10.66	465 [M+H]^+^	303(100)	Quercetin hexoside	83.7%
**21**	11.04	595 [M+H]^+^	271(8) 287(100) 433(15) 449(41)	Kaempferol-glucorhamnoside	85.9%
**22**	11.3	268	84(100) 227(5)	UI	
**23**	11.51	609 [M+H]^+^	303(100) 345(15) 411(5) 573(8) 591(13)	Quercetin 3-[6’’-(3-hydroxy-3-methylglutaryl)galactoside]	91.0%
**24**	11.68	695	488(7) 506(9) 520(17) 538(5) 627(9) 641(6) 645(77) 659(6) 663(11) 677(100)	UI	
**25**	11.64	595 [M+H]^+^	287(100) 449(20)	Kaempferol-glucorhamnoside (isomer)	97.0%
**26**	11.91	923 [M-OH]^+^	279(11) 305(100) 413(27) 431(13) 431(67) 456(9) 457(22) 565(5) 583(8) 583(21) 601(14) 735(10) 753(12) 771(33)	Pentagalloylglucopyranose	
**27**	12.20	435 [M+H]^+^	303(100)	Quercetin pentoside	97.3%
**28**	12.69	449 [M+H]^+^	287(100)	Trifolin	91.7%
**29**	13.05	1075 [M-OH]^+^	395(5) 413(18) 431(12) 431(44) 457(60) 565(11) 583(21) 601(10) 609(18) 717(5) 735(26) 753(16) 771(12) 887(13) 905(16) 923(100)	Hexagalloylglucopyranose	See comment
**30**	13.20	1227 [M-OH]^+^	583(20) 735(29) 923(20) 1075(100)	Heptagalloylglucopyranose	See comment
**31**	13.28	593	287(100) 329(12) 395(8) 557(9) 575(21)	UI	See comment
**32**	13.33	419 [M+H]^+^	287(100) 383(5)	Juglanin	91.7%
**33**	13.89	419 [M+H]^+^	287(100) 383(5) 401(15)	Juglanin (isomer)	87.2%
**34**	14.01	679	490(6) 504(7) 611(7) 629(9) 629(53) 643(7) 647(26) 661(100)	UI	
**35**	14.23	433 [M+H]^+^	271(21) 287(100) 397(5)	Kaempferol-3-*O*-β-rhamnoside	79.8%
**36**	14.84	491	287(100) 395(7) 473(5)	UI	See comments
**37**	15.11	515	161(11) 175(100) 193(22) 318(5) 336(36) 353(34) 498(18)	UI	
**38**	16.22	475	457(100) 439(15) 287(15) 312(5) 275(5)	UI	
**39**	16.38	287 [M+H]^+^	121(16) 133(12) 135(15) 145(12) 153(100) 161(11) 165(53) 197(12) 213(48) 231(25) 241(72) 245(18) 258(28) 259(20) 269(17)	Kaempferol	89.7%

Unidentified compound (UI). The percentage indicates the score reported in the MzCloud database.

In addition, the chemical analysis of the four fractions revealed the presence of phenolic acids (IDs 14, 16, and 17 in Table 6), numerous flavonoids (mostly quercetin derivatives, IDs 18, 20, 23, and 27, and kaempferol derivatives, IDs 21, 25, 28, 32, 33, 35, and 39), and gallotannins (IDs 15, 19, 26, 29, and 30). The clustering of the identified compounds indicated four major groups (Fig 5B): The first group (cluster 1; Fig 5B in blue) is composed mainly of monoglycosylated quercetin (IDs 20 and 27) and kaempferol (IDs 28, 32, 33, and 35) derivatives. The second group (cluster 2; Fig 5B in light blue) is comprised of di-glycosylated derivatives of quercetin (ID18) and kaempferol (IDs 21 and 25), as well as 3-[6’’-(3-hydroxy-3-methylglutaryl)galactoside] quercetin (ID23) and di-glycosylated pelargonidin (ID8). The third group (cluster 3; Fig 5B in green) consists of amino acids (ID10), sugar (ID2), kaempferol (ID39), and phenolic acids (IDs 14, 16, and 17). The fourth group (cluster 4; Fig 5B in orange) has metabolites with a complex structure, consisting mainly of gallotannins (IDs 15, 19, 26, 29, and 30).

Fig 5C shows the average relative abundance computed by the normalised peak areas of each of the identified compounds in each cluster. Additionally, we add the mean relative abundance of the unidentified peaks (defined as cluster 5). All average relative abundance of the clusters decreases after fraction 4 (as we would expect, based solely on the solubility gradient). However, in fraction 3, we can see the highest abundance of clusters 1, 2, 3, 5, and, to a lesser degree, 4. The relative abundance composition in fractions 1 and 2 is fairly similar, but an increment is noticed in fractions 3 and 4. The increment of relative abundance composition noticed in fraction 3 may be responsible for its slightly better inhibitory activity (Fig 4B) when compared with fractions 1, 2, and 4.

Numerous studies point towards the synergistic action of polyphenols in combating microbial growth [72–76]. This synergistic effect arises from the intricate interplay between various secondary metabolites within natural extracts. These interactions, influenced by both polyphenol structure and environmental factors, modulate the overall biological activity [72]. Notably, polyphenols possess the ability to affect multiple biological targets, including enzymes, transport proteins, membrane receptors, and lipid membranes, among others [73]. In fact, a study realised by Mokhar and colleagues demonstrated the antimicrobial activity of polyphenols present in pepper (*Capsicum annuum L*.) against *S*. *aureus* ATCC 6538, evidencing the individual efficacy of quercetin and kaempferol compounds. Moreover, when combined with caffeic acid, these compounds also showed a synergistic effect [74]. Other examples of synergistic effects with polyphenols have been reported, such as combinations of rutin with quercetin, morin, kaempferol, myricetin, and eriodictyol against *Bacillus cereus*, despite the fact that rutin alone does not possess antibacterial activity [75]. Therefore, the synergistic effects appear to depend on specific ratios and combinations of polyphenols [76].

In our particular case, the antibacterial activity noted on fractions 1 to 4 against *S*. *aureus* ATCC 25923 could be attributed mainly to the presence of flavonoids such as *quercetin* derivatives (e.g. quercetin hexoside, quercetin pentoside, rutin) and *kaempferol* and its derivatives (e.g. kaempferol-glucorhamnoside, trifolin, juglanin, and kaempferol-3-*O*-β-rhamnoside), and their synergistic interaction with *gallotannins* (e.g. 1,2,6-trigalloylglucose, tetragalloylglucopyranose, pentagalloylglucopyranose, hexagalloylglucopyranose, and heptagalloylglucopyranose) and *anthocyanins* (e.g. cyanidin 3,5-diglucoside, pelargonidin-galactoside, pelargonidin, 3,5-diglucoside and peonin). However, the increase of concentration in cluster 5 (unidentified molecules) in fractions 3 and 4 may indicate that some of the molecules in this cluster may also have a potential antibacterial effect or a synergistic effect in combination with the secondary metabolites mentioned above, which is enhanced by their increased concentration.

Overall, few studies evaluated the biological activity of pure compounds, and most of the reports focus on the antibacterial activity of crude extracts containing a mix of compounds. For instance, a study using pure quercetin compounds exhibited selective antibacterial properties towards *S*. *aureus*, MRSA, and *S*. *epidermidis* [77]. In addition, quercetin pentoside and other quercetin derivatives identified in walnut leaves were found to selectively inhibit the growth of Gram-positive bacteria (*B*. cereus, *B*. *subtilis*, S. aureus) [78]. Likewise, rutin (a quercetin derivative) exhibited MIC values of 512 μg/mL and 256 μg/mL towards *S*. *aureus* ATCC 25923 and *E*. *faecalis* ATCC 29212 [79] respectively.

In addition, pure kaempferol and kaempferol-containing extracts have exhibited antibacterial activity towards clinically important bacterial pathogens such as *S*. *aureus* and *E*. *faecalis* [80]. Antibacterial activity towards *S*. *aureus* and MRSA strains have also been reported in studies with different plant extracts containing kaempferol derivatives such as trifolin (kaempferol 3-*O*-β-D- galactopyranoside) in *Terminalia petiolaris* methanolic extract [81] and kaempferol-3-rhamnoside in the ethyl acetate extract of *Zanthoxylum bungeanum* leaves [82]. Nonetheless, other polyphenols were also identified in these plant extracts that could also be associated with the antimicrobial activity reported.

Finally, gallotannins are characterised by the presence of galloyl groups, which are reported to be responsible for gallotannins’ higher antibacterial activity compared to other hydrolysable tannins [58]. In the present study, we identified different gallotannins with a variety of galloyl groups, which may explain the best antibacterial activity encountered in *P*. *zonale* extract when compared to *P*. *graveolens* and *P*. *odoratissimum*.

Thus, based on this information, the antibacterial activity reported for *P*. *zonale* towards the Gram-positive bacteria *S*. *aureus* ATCC 25923, MRSA 333, *E*. *faecalis* ATCC 29212, and Vancomycin-resistant *E*. *faecalis* INSPI 032 (Table 5) may be attributed to the presence of these compounds. Although no specific metabolites were identified among the three species, *P*. *zonale* distinguishes itself notably for containing gallotannins and anthocyanins, which are unique to this species and may interact synergistically with other metabolites present.

In summary, our study evaluated the chemical profiles of three distinct species within the *Pelargonium* genus. Our findings revealed the presence of quercetin and kaempferol derivatives in *P*. *graveolens*, *P*. *odoratissimum*, and *P*. *zonale*. Notably, gallotannins and anthocyanins were uniquely identified in *P*. *zonale*. Likewise, *P*. *graveolens* stands out due to the various types of myricetin derivatives that were not detected in *P*. *odoratissimum* and *P*. *zonale*. Furthermore, *P*. *zonale* displayed superior antibacterial and antibiofilm activities in comparison to the other two species. The antibacterial activity of *P*. *zonale* was observed towards the clinically relevant strains of *S*. *aureus* ATCC 25923, MRSA 333, *E*. *faecalis* ATCC 29212, and Vancomycin-resistant *E*. *faecalis* INSPI 032. Fractionation analysis of *P*. *zonale* suggests that the antibacterial activity attributed to this plant is due to the presence of quercetin derivatives and kaempferol and its derivates, alongside their synergistic interaction with gallotannins and anthocyanins. Given the current challenges posed by the depletion of effective drug options against the resistant strains of *S*. *aureus* and *E*. *faecalis*, the secondary metabolites encountered in the fragmentation of *P*. *zonale* deserve further investigation.

## Supporting information

S1 Fig Time-kill curves. (DOCX)

S2 Fig The chemical characterisation of fractions 1 to 4. (DOCX)

S1 Table Chemical identification explanation with corresponding figures. (DOCX)

S2 Table Biofilm inhibition and eradication. (DOCX)

S3 Table Chemical identification explanation of fractionation. (DOCX)
