# Supplementary material for: A chemical analysis of the Pelargonium species: P. odoratissimum, P. graveolens, and P. zonale identifies secondary metabolites with activity against gram-positive bacteria with multidrug-resistance
Source: PLoS One. 2024 Jul 10;19(7):e0306637. doi: 10.1371/journal.pone.0306637 (PMC11236107; doi:10.1371/journal.pone.0306637)
Supplement: S1 Fig — (DOCX) [file pone.0306637.s001.docx]

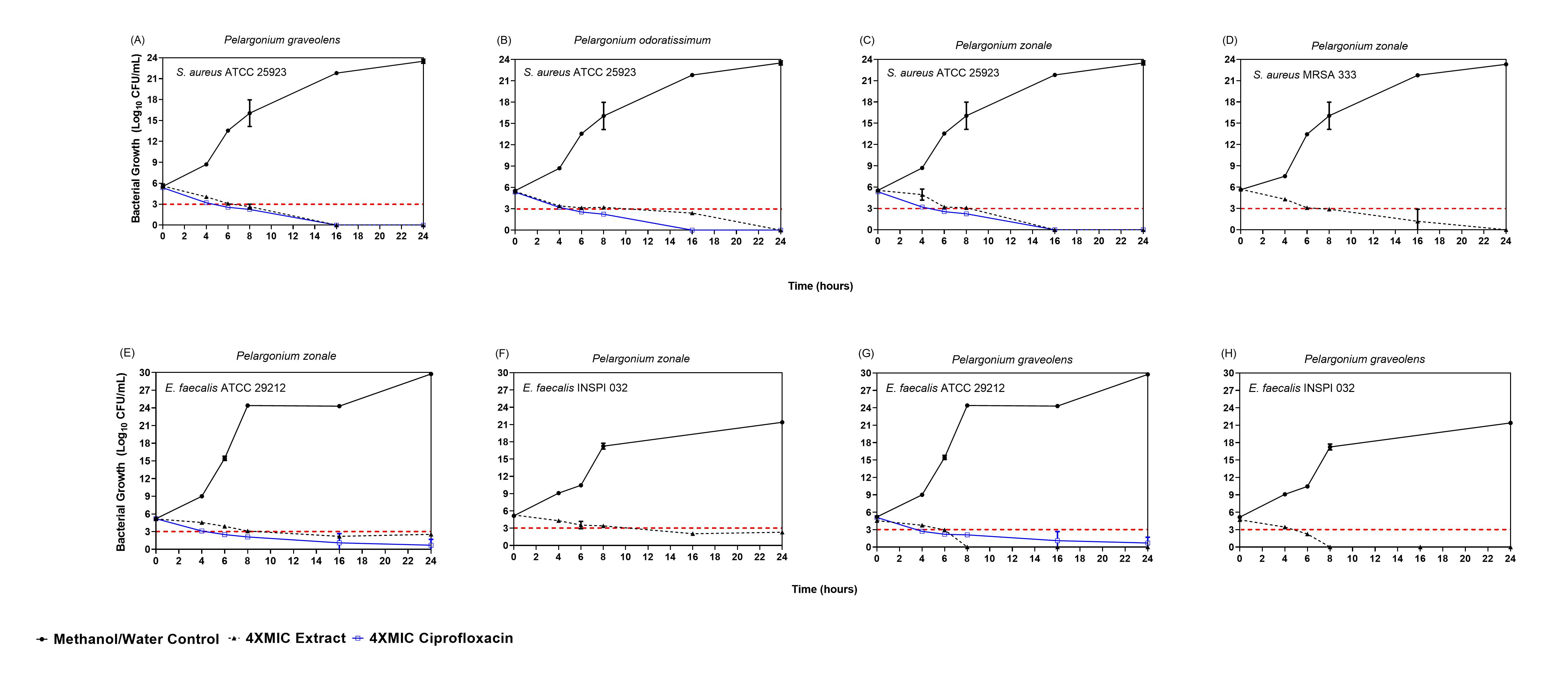


**Supplementary Figure 1.** Time–kill curves of the methanolic extracts of *P. graveolens*, *P. odoratissimum* and *P. zonale* against *S. aureus* ATCC 25923, MRSA 333, *E. faecalis* ATCC 29212, or the Vancomycin-Resistant *E. faecalis* INSPI 032. The concentration of the methanolic extracts (black triangle) and the positive control ciprofloxacin (blue square) is 4X MIC. The negative control (black circle) was a solution with a concentration of methanol-water (80:20, v/v). The horizontal red line symbolizes the limit for bactericide activity (≥ 3-log_10_ decrease). Error bars indicate ± SD of two independent experiments.
