# Supplementary material for: A chemical analysis of the Pelargonium species: P. odoratissimum, P. graveolens, and P. zonale identifies secondary metabolites with activity against gram-positive bacteria with multidrug-resistance
Source: PLoS One. 2024 Jul 10;19(7):e0306637. doi: 10.1371/journal.pone.0306637 (PMC11236107; doi:10.1371/journal.pone.0306637)
Supplement: S2 Fig — (DOCX) [file pone.0306637.s002.docx]

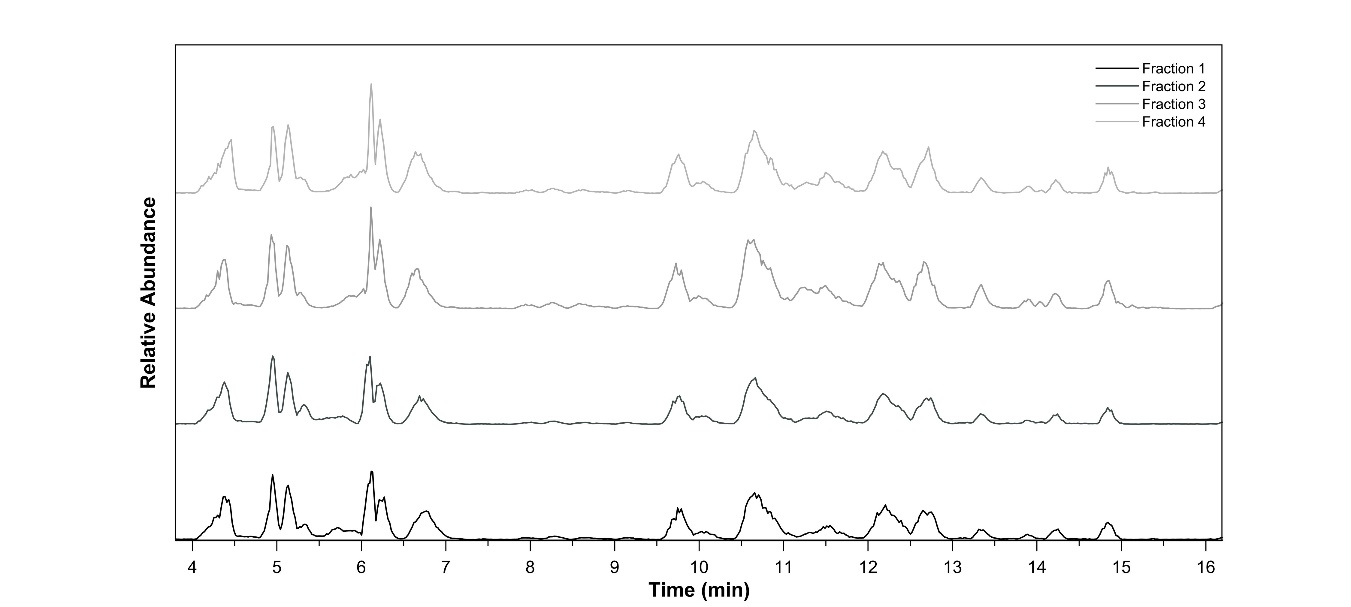


**Supplementary Figure 2A.** Chemical analysis of the fractions 1 to 4. HPLC-DAD of fractions 1, 2, 3 and 4 of *P. zonale* obtained after separation by solid phase extraction.


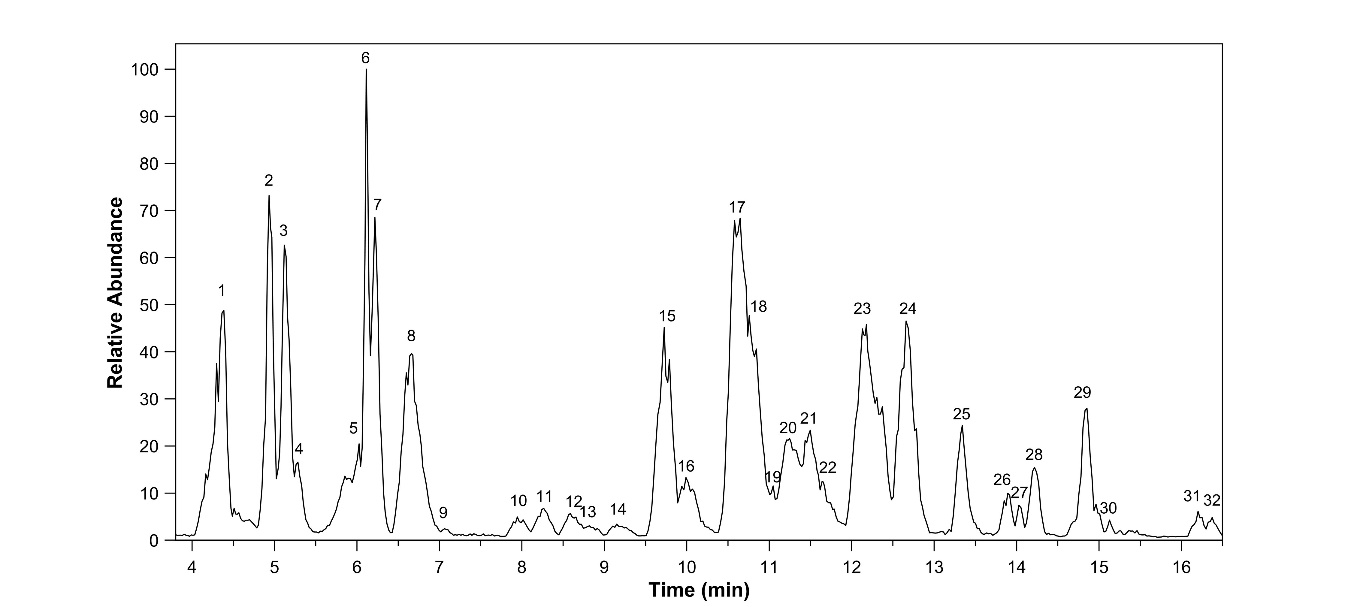


**Supplementary Figure 2B.** Representative HPLC-DAD chromatogram of fractions 1 - 4 from *P. zonale* after solid-phase extraction.
