# Supplementary material for: A chemical analysis of the Pelargonium species: P. odoratissimum, P. graveolens, and P. zonale identifies secondary metabolites with activity against gram-positive bacteria with multidrug-resistance
Source: PLoS One. 2024 Jul 10;19(7):e0306637. doi: 10.1371/journal.pone.0306637 (PMC11236107; doi:10.1371/journal.pone.0306637)
Supplement: S1 Table — (DOCX) [file pone.0306637.s003.docx]

**Supplementary Table 1.** Tentative identification of the most abundant masses in *Pelargonium* species extracts HPLC - DAD - MS/MS.

| ID | *P. g* | *P. o* | *P. z* | Parent mass  *m/z* | Main MS/MS products (*m/z*) | Tentative identification | References |
| --- | --- | --- | --- | --- | --- | --- | --- |
|  | **Rt (min)** | | |  |  |  |  |
| 1 |  |  | 1.15 | 381  [M+K]^+^ | 201(100) 219(90) 249(25) 363(25) 207(25) 235(25) | Sucrose | (Overy et al. 2008) |
| 2 |  |  | 1.53 | 308  [M+H]^+^ | 179(100) 162(25) 233(15) 290(5) | L-Glutathione | 89.8% |
| 3 |  |  | 2.35 | 322  [M+H]^+^ | 193(100) 176(20) 233(10) 304(5) | L-l-Homoglutathione | (Ramos-Escudero et al. 2010) |
| 4 | 4.98 | 4.90 |  | 611  [M+H]^+^ | 443(100), 317(30) 425(20) 307(10) 287(5) | A type of (epi)-gallocatechin dimer | (Liu, Kallio, and Yang 2014; Shui, Wong, and Leong 2004) |
| 5 |  |  | 5.58 | 678  [M+H]+ | 354(100) 516(80) | UI |  |
| 6 |  | 6.23 |  | 220  [M+H]^+^ | 202(100) 90(40) 184(30) | Pantothenic acid | 90.0% |
| 7 |  |  | 6.68 | 205  [M+H]^+^ | 188(100) | DL-tryptophan | 91.9% |
| 8 | 5.61 | 6.81 |  | 307  [M+H]^+^ | 139(100) 289(95) 150(80) 181(10) | (-)-Epigallocatechin | 92.7% |
| 9 | 6.96 | 7.41 |  | 205  [M+H]^+^ | 188(100), 159 (5) | DL-Tryptophan | 90.9% |
| 10 | 8.45 |  |  | 1219  [M+H]^+^ | 915(100) 1051(30) 895(30) 1093(10) 1134(10) 757(5) 699(5) 611(5) | A type of (epi)-gallocatechin tetramer | (Liu et al. 2014) |
| 11 | 8.55 |  |  | 305  [M+H]^+^ | 287(100) 127(70) 143(60) 277(50) 179(20) | UI | See comments |
| 12 | 8.20 | 9.86 |  | 611  [M+H]^+^ | 425(100) 287(87) 443(82) 317(61) 307(55) 305(34) | A type of (epi)-gallocatechin dimer | (Liu et al. 2014) |
| 13 | 9.11 | 9.67 |  | 915  [M+H]^+^ | 609(100) 611(75) 747(25) 836(20) | A type of (epi)-gallocatechin trimer | (Liu et al. 2014) |
| 14 |  |  | 9.44 | 395  [M+H]^+^ | 233(100) 377(35) 246(25) 215(10) 132(10) | UI | UI |
| 15 | 9.46 |  |  | 1523  [M+H]^+^ | 1183(100) 895(95) 1216(80) 851(70) 1016(60) | A type of (epi)-gallocatechin pentamer | (Shui et al. 2004) |
| 16 |  | 9.61 |  | 915  [M+H] | 747(100) 609(80) 739(40) 878 (25) 611(35) | A type of (epi)-gallocatechin trimer | (Liu et al. 2014) |
| 17 |  |  | 9.66 | 611  [M+H]^+^ | 287(100) 449(75) | Cyanidin 3,5-diglucoside | (Macz-Pop et al. 2006) |
| 18 |  | 9.81 |  | 295  [M+H]^+^ | 166(100) 120(20) | Gamma-glutamylphenylalanine | MassBank Record: MSBNK-Metabolon-MT000057 |
| 19 | 10.18 |  |  | 291  [M+H]^+^ | 123(100) 139(80) 273(60) 165(40) 245(30) | Catechin | 80.8% |
| 20 |  |  | 10.3 | 595  [M]^+^ | 433(100) 271(98) 576(14) 325(5) | Pelargonidin 3,5-diglucoside | (Macz-Pop et al. 2006) |
| 21 | 10.34 |  |  | 344  [M+H]^+^ | 165(100) 147(50) 309(20) 180(10) | 4-*O*-coumaroyl–amino glycoside | (Lozovaya et al. 2007) |
| 22 |  |  | 10.65 | 625  [M]^+^ | 463(100) 301(80) 286(5) | Peonin | (Ramos-Escudero et al. 2010) |
| 23 | 10.98 | 11.17 |  | 389  [M+H]^+^ | 209(100) 227(30) 371(10) 191(10) | 4-[4-hydroxy-2,6,6-trimethyl-3-[3,4,5-trihydroxy-6-(hydroxymethyl)oxan-2-yl]oxycyclohexen-1-yl]butan-2-one | 82.2% |
| 24 |  |  | 11.93 | 339  [M+H]^+^ | 147(100) 321(5) 119(5) | Coumaryl quinic acid | 78.1% |
| 25 | 12.18 |  |  | 393  [M+H]^+^ | 197(100) 375(60) 285(40) 345(20) | UI |  |
| 26 |  |  | 12.18 | 433  [M+H]^+^ | 271(100) | Pelargonidin galactoside | (da Silva et al. 2007) |
| 27 |  |  | 12.39 | 611  [M+H]^+^ | 449(100) 287(25) | Luteolin 3',4'*-O*-beta-D-glucopyranoside | 81.0% |
| 28 | 12.91 |  |  | 613  [M+H]^+^ | 319(100) 481(50) 595(20) | Myricetin 3-sambubioside | See comments |
| 29 |  | 12.91 |  | 502  [M+H]^+^ | 373(100) 355(50) 209(10) 484(10) | N5-((S)-1-((carboxymethyl)amino)-3-(((R)-1-(3,4-dimethoxyphenyl)-3-hydroxypropan-2-yl)thio)-1-oxopropan-2-yl)-L-glutamine | (Yao, Peng, and Zheng 2016) |
| 30 | 13.09 |  |  | 369  [M+H]^+^ | 193(100) 235(50) 351(50) 333(40) 176(25) 215(25) 277(20) | UI |  |
| 31 |  |  | 13.24 | 595  [M+H]^+^ | 449(100) 287(30) 576(20) 433(20) | UI |  |
| 32 | 13.3 |  | 13.29 | 627  [M+H]^+^ | 303(100) 465(80) 319(30) 609(10) | Quercetin 3,4'-diglucoside | (Häkkinen and Auriola 1998) |
| 33 | 13.48 |  |  | 481  [M+H]^+^ | 319(100) 462(5) | Myricetin 3-*O*-beta-D-galactopiranose | 90.3% |
| 34 | 13.83 |  |  | 597  [M+H]^+^ | 303(100) 465(50) 435(10) | Quercetin-3-*O*-vicianoside | 79.4% |
| 35 | 14.39 |  |  | 451  [M+H]^+^ | 319(100) 329(5) | Myricetin pentoside | See comments |
| 36 | 14.47 | 14.52 | 14.42 | 611  [M+H]^+^ | 303(100) 465(30) | Rutin | 89.8% |
| 37 | 14.68 |  | 14.67 | 465  [M+H]^+^ | 303(100) | Quercetin hexoside | See comments |
| 38 |  |  | 14.78 | 595  [M+H]^+^ | 287(100) 449(40) 433(10) 576(5) | Kaempferol glucorhamnoside | 91.0% |
| 39 |  | 14.96 |  | 433  [M+H]^+^ | 415(100), 367(50), 271(40), 397(25), 337(20), 313(20), 379(15) | Vitexin | 87.3% |
| 40 | 15.36 |  | 15.95 | 595  [M+H]^+^ | 287(100) 449(30) 576(5) | Kaempferol glucorahamnoside  (isomer 1) | 97.6% |
| 41 | 15.63 |  | 15.47 | 435  [M+H]^+^ | 303(100) | Quercetin pentoside | See comments |
| 42 |  | 15.71 |  | 437  [M+H]^+^ | 419(100) 341(30) 383(25) 317(25) 401(15) | p-Phlorizin | 87.1% |
| 43 | 15.98 |  |  | 373  [M+H]^+^ | 211(100) 193(20) 355(20) 175(10) 135(10) | 4-hydroxy-5-methoxy-trans-melilotoside | (Yang et al. 2007) |
| 44 | 16.02 |  |  | 625  [M+H]^+^ | 317(100) 479(25) 593(5) | Rhamnetin-3-rutinoside | 78.1% |
| 45 | 16.17 | 16.26 |  | 449  [M+H]^+^ | 287(100) 430(5) | Trifolin | 89.9% |
| 46 | 16.58 |  | 16.38 | 419  [M+H]^+^ | 287(100) 401(5) | Juglanin | 87.0% |
| 47 | 17.12 |  |  | 275  [M+H]^+^ | 107(100) 149(50) 169(30) 127(20) | Phloretin | 87.1% |
| 48 | 17.30 |  |  | 679  [M+H]^+^ | 661(100) 647(80) 623(30) 522(10) | Ov-NCC-1 | (Müller et al. 2011) |
| 49 |  |  | 18.09 | 491  [M+H]^+^ | 303(100) 189(90) 473(60) 459(20) | Quercetin 3-*O*-acetyl-rhamnoside | FooDB database: compound FDB000163 |

Unidentified compound (UI). The percentage indicates the score reported in the MzCloud database. *P.g*: *Pelargonium graveolens*, *P.o*: *P. odoratissimum* and *P.z*: *P. zonale.* FooDB accessible at: https://foodb.ca/

Identification (ID) numbers 1, 2, and 3 were characterised based on the rupture pattern of *P. zonale.* The parent mass 381 *m/z* (ID1) was previously identified as sucrose, a disaccharide, with an [M+K]+ ion (Overy et al. 2008). The product ions at 201 and 219 *m/z* correspond to the monosaccharides (hexose) [M(mono) + K]+ and [M(mono) + K - H2O]+, respectively**. The best match score for the parent mass of 308 *m/z* (ID2) in MzCloud was the protonated L-glutathione, and the product ions of this mass were 179, 219, and 249 *m/z*. These ions correspond to losses of** .**g-glutamic acid (-129 Da and -146 Da) and glycine (-75 Da)**(Leblanc and Sleno 2011). **The identification of the compound corresponding to mass 322 *m/z* (ID3) was previously described as L-l-homoglutation (Oven et al. 2001). In addition, in *P. zonale,* a parent mass of 678 *m/z* (ID5) with a primary rupture at 516 and 354 *m/z* was detected. These** fragmentation patterns indicate the loss of one and two hexoses, respectively, but no further rupture of 354 *m/z* was noticed.


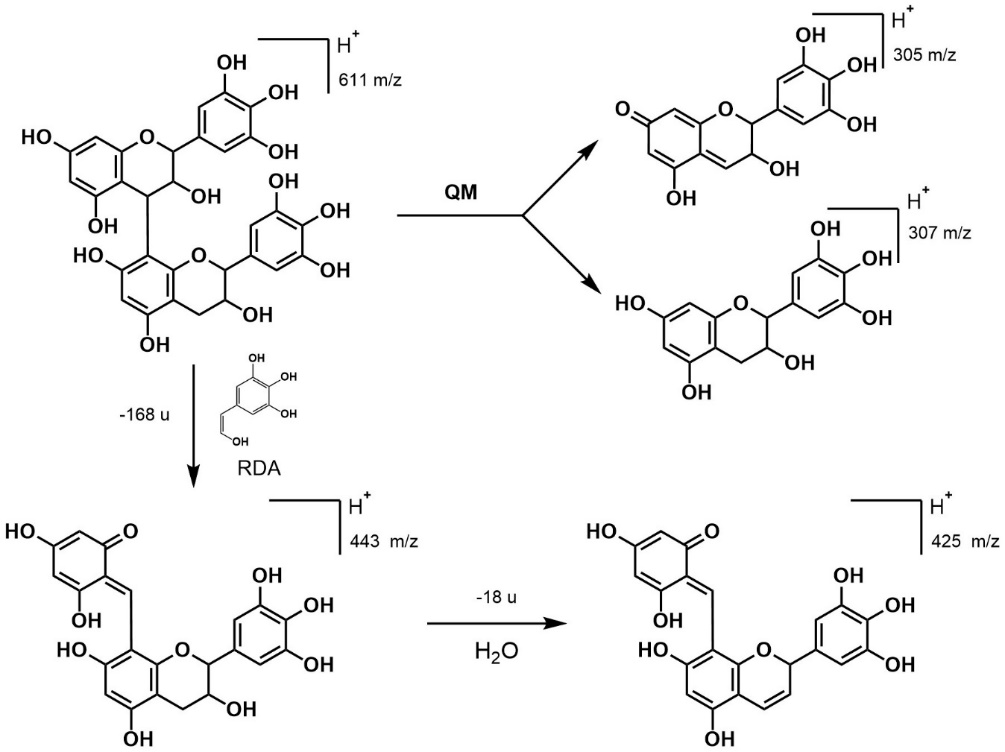


Figure S1-1. Fragmentation patterns of the gallocatechin dimer (611 *m/z*)

The parent mass 611 *m/z* (ID4 and ID12) was detected with retention times of 4.98 and 4.90 min in both *P. graveolens* and *P. odoratissimum*, exhibiting a scission pattern analogous to the previously described for gallocatechin dimers (Liu et al. 2014)(Shui et al. 2004). The ion fragmented at 443, 317 *m/z*, 425 *m/z* and 307 *m/z* in ID4, and at 425 *m/z*, 287 *m/z*, 443 *m/z*, 317 *m/z*, 307 *m/z* and 305 *m/z* in ID12. Figure S1-1 illustrates the proposed fragmentation mechanism for gallocatechin dimers. In this pattern, the fragment ions at *m/z* 443 [M + H - 168]+ could be attributed to retro-Diels-Alder (RDA) fission cleavage of the heterocyclic rings, while the ion at *m/z* 425 could be the result of water loss from the ion at *m/z* 443. On the other hand, the ions at *m/z* 305 and 307 are quinone-methide (QM) fission fragments, resulting from the cleavage of the interflavane bond between the (epi)-gallocatechin monomers. Neutral losses of 304 and 306 uma are observed for the top and terminal units, respectively. These cleavage patterns have been observed and described previously for oligomeric proanthocyanidins (Engström et al. 2014) and prodelphinidins (Callemien and Collin 2008).


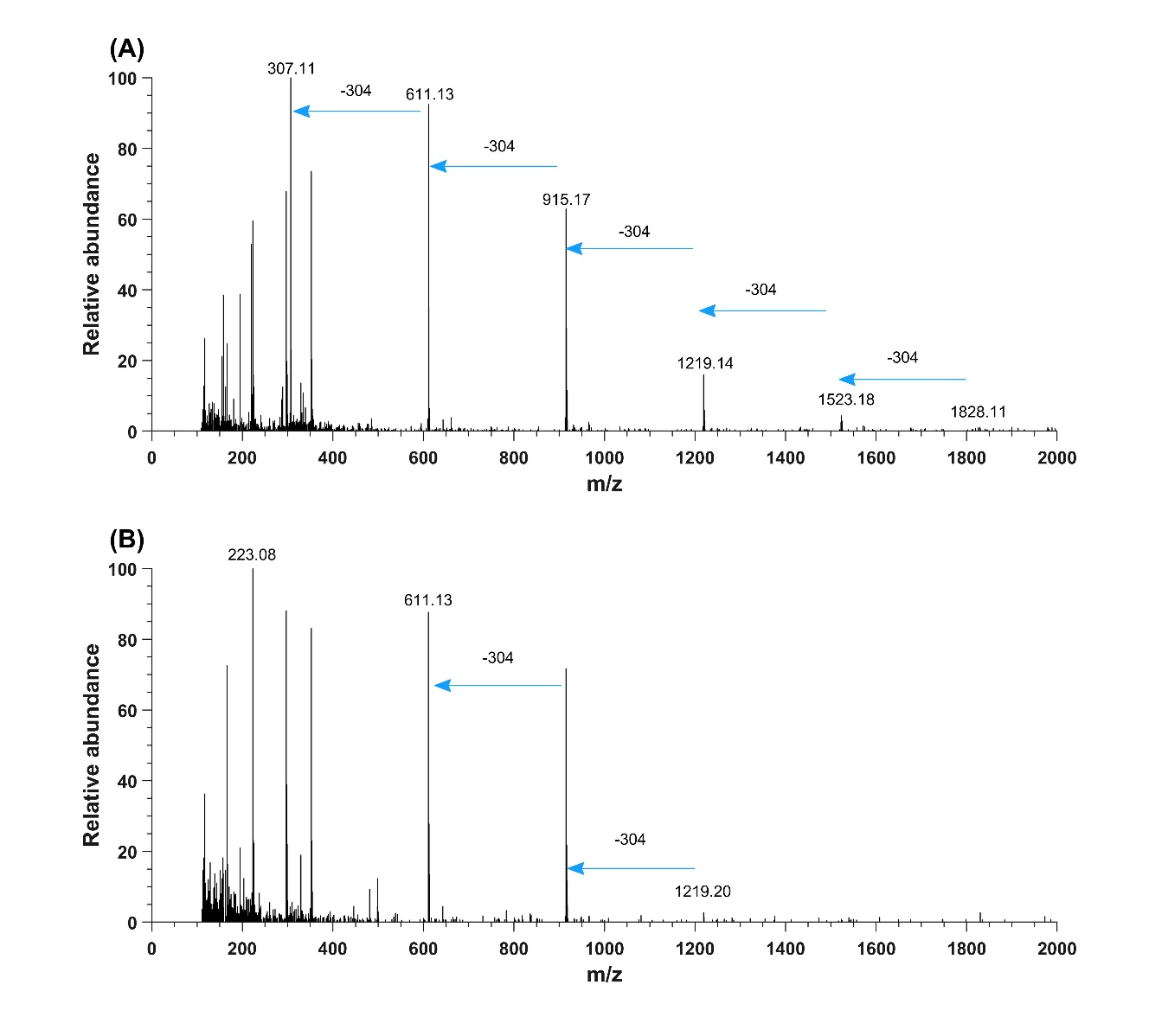


Figure S1-2. Mass spectrum representation of polymers of gallocatechin in (A) *P. graveolens* at = 4.98 *min* and (B) *P. odoratissimum* at RT = 4.90 min.

Furthermore, further QM fragmentation can occur for oligomers comprising three or more monomeric units due to repeated cleavage of the interflavonoid bonds connecting the extension units. These fragmentation products are also employed for the identification of such compounds (Lin et al. 2014). In the case of *P. graveolens*, additional mass ions were also identified, namely 915 (ID13), 1219 (ID10) and 1523 (ID15) *m/z*, which appeared to be very close to the 611 *m/z* parent mass. Similarly, the 915 and 1219 ions were observed in *P. odoratissimum*, as shown in Figure S1-2. These masses demonstrate a consistent and consecutive loss of 304 uma, corresponding to a QM fragmentation type, as illustrated in Figure S1-3, which depicts the fragmentation pattern of *P. odoratissimum*. Consequently, these masses are consistent with various degrees of polymerisation of gallocatechin, as previously reported by Liu et al. (2014). These ions are also identified in *P. graveolens* in the retention time range between 7.7 min and 9.75 min. Furthermore, a variation in the intensities of these ions is observed, with 1219 *m/z* being the most prominent at 8.45 min, 915 *m/z* at 9.11 min, and 1523 *m/z* at 9.46 min. The consistency between the spectral masses and the neutral loss of 304 suggests the presence of (epi)-gallocatechin polymers at these retention times, with no other structure being present. In Table 3 in the mian manuscript, we employed the term "A type of (epi)-gallocatechin" because these oligomers may be gallocatechin, epigallocatechin or a mixture of both to indicate the uncertainty of the exact combination presented. Previous studies have identified gallocatechin polymers in *P. graveolens* hydroethanolic extracts (Al-Sayed et al. 2015) and by us in *P. odoratissimum* (Fernandez-Soto et al. 2023). Moreover, the mass with an *m/z* value of 307 (ID8) was successfully identified in *P. graveolens* and *P. odoratissimum* species as (-)-epi-gallocatechin with a high MzCloud score of 92.7%. This compound exhibits a predominant fragment ion at 139 *m/z*, which is characteristic of catechins and corresponds to a Retro-Diels-Alder (RDA) fragment (Hokkanen et al. 2009). Additionally, the fragment ion at 289 *m/z* is indicative of a water loss. Finally, in ID11, we found a molecular ion at 305 *m/z*, described as monomeric products from the cleavage of gallocatechin polymers (Liu et al. 2014), as we can see in Figure S1-2 with product ions at 287, 127, 143, 277, and 179 *m/z*.


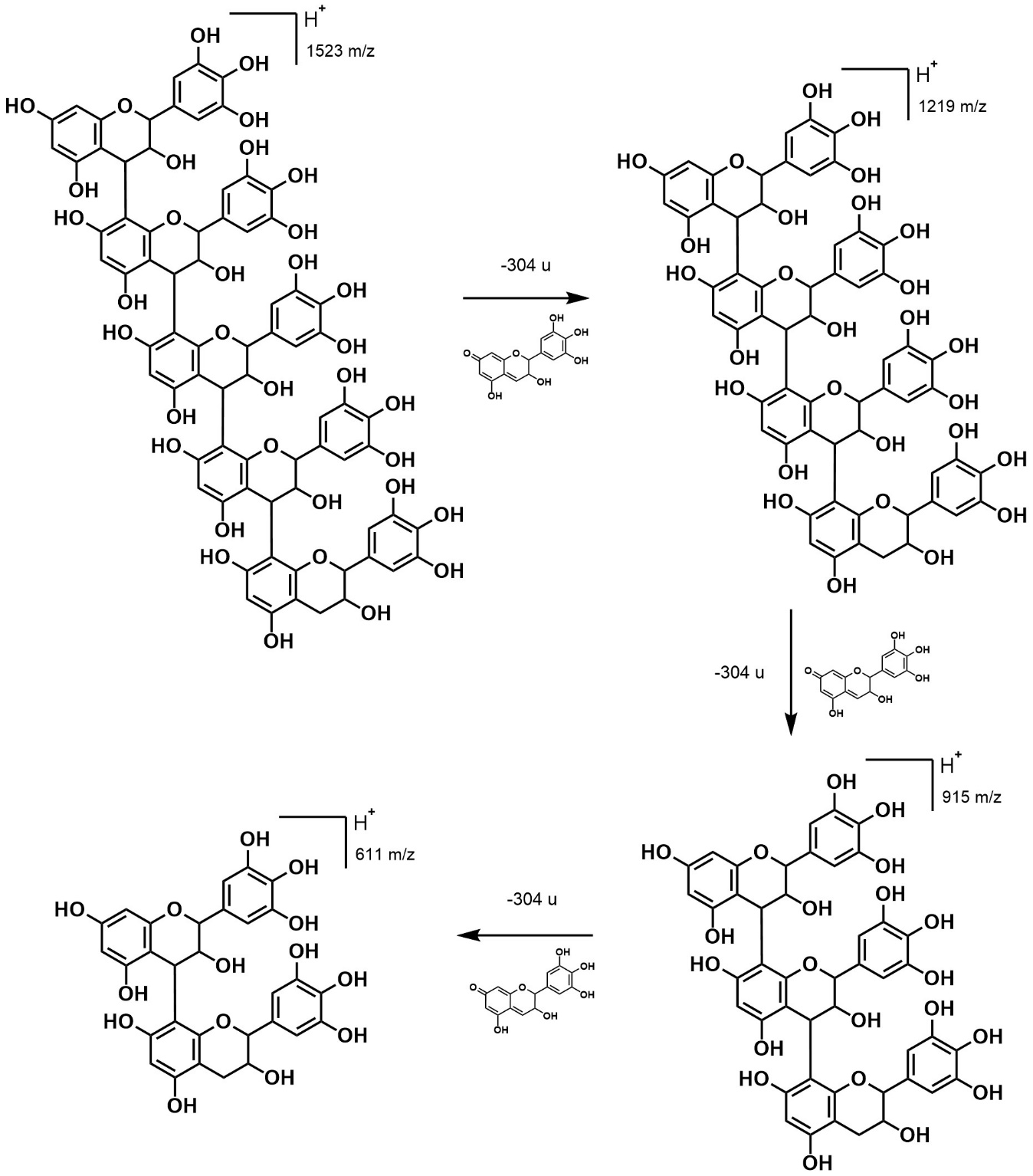


Figure S1-3. Structures of Gallocatechin Oligomers: Dimer (611 *m/z*), Trimer (915 *m/z*), Tetramer (1219 *m/z*), and Pentamer (1523 *m/z*)

The mass fragmentation pattern of the ID6 detected in *P. zonale* matched that of pantothenic acid with a parent mass of 220 *m/z* and a score of 90.0% in MzCloud. The mass differences of 220 - 202 (18 Da) and 220 - 184 (36 Da) are consistent with the neutral loss of one and two water molecules, while 90 *m/z* is consistent with the formation of protonated alanine. The parent mass of 205 *m/z* (ID 7 and ID9) is presented in the three *Pelargonium* species and gave the best match score of 91.9% in MzCloud as DL-tryptophan. The mass pattern includes a dominant ion at 188 *m/z* [M-NH3]+. There is also a less intense peak at 159 *m/z* [M-H2O-CO]+.

The compound with a parent mass of 611 *m/z* (ID 17) with an [M]+ ion was previously identified as cyanidin 3,5-diglucoside (Macz-Pop et al. 2006). This mass has two fragments at *m/z* 287 and 449, which would correspond to the losses of one and two hexoses, respectively. The masses 595 (ID20) and 433 (ID26) *m/z* have a fragment at 271 *m/z*, consistent with the presence of pelargonidin (Macz-Pop et al. 2006)(da Silva et al. 2007). The fragmentation pattern of 625 *m/z* (ID22) exhibits a significant peak at 301 *m/z*, indicating the presence of peonidin aglycone. Additionally, a minor fragment at 463 *m/z* corresponds to a neutral loss of 162 Da, suggesting the presence of a hexoside moiety. Another author has also reported a mass of 625 *m/z* as peonin (Ramos-Escudero et al. 2010). Importantly, these four anthocyanins: cyanidin 3,5-diglucoside, pelargonidin 3,5-diglucoside, peonin, and pelargonidin galactoside, are only present in *P. zonale*, which explains the anthocyanin content of this plant (Table 2, main manuscript).

The parent mass 344 *m/z* (ID21) detected in *P. odoratissimum* produces two major ions at 165 and 147 *m/z*, this compound was preliminarily identified as trans-p-Coumaric acid 4-glucoside with a score of 78.6% in MzCloud. However, the molecular mass of this compound is 326 Da, which is 18 Da less than that of the parent ion. The 344 *m/z* ion with fragmentations at 325, 165, and 147 *m/z* was previously identified and attributed to a 4-*O*-coumaroyl-amino glycoside (Lozovaya et al. 2007). The presence of the 325 *m/z* ion is not observed in our MS-MS spectrum. The mass difference of 344 - 325 (17 Da) is consistent with the loss of an amine group, which we were not able to identify. Nevertheless, we believe that our compound may be a 4-*O*-coumaroyl-amino glycoside due to the coincidence of the parent ion and the two daughter ions. For the parent mass of 339 *m/z* (ID24) the best match (score 78.1%) in MzCloud was identified as coumaroylquinic acid in *P. zonale*. This mass pattern is characterized by a dominant ion at 147 *m/z* [M+H-quinic acid]+ and a lower intensity peak at 119 *m/z* [M+H-quinic acid-CO]+. The parent mass 502 *m/z* (ID29) identified in *P. odoratissimum* produced fragmentations consistent with the masses of N5-((S)-1-((carboxymethyl)amino)-3-(((R)-1-(3,4-dimethoxyphenyl)-3-hydroxypropan-2-yl)thio)-1-oxopropan-2-yl)-L-glutamine previously reported by Yao and colleagues (Yao et al. 2016). However, the ion intensities differ considerably, which may be related to the distinct experimental conditions employed.

The molecular ion at *m/z* 613 (ID28) produces fragments at *m/z* 319 and 481. The loss of sambubioside (294 Da) linked to myricetin results in the formation of the ion at *m/z* 319, while the loss of xyloside (132 Da) from the sambubioside moiety generates the ion at *m/z* 481. This fragmentation pattern aligns with the structure of myricetin *O*-sambubioside. The compound with a molecular ion at *m/z* 481 (ID33) also produces a fragment at *m/z* 319. This ion was identified in MzCloud as myricetin 3-*O*-β-D-galactopyranose (score 90.3% in MzCloud). The mass difference of 481 - 319 *m/z* corresponds to a hexoside moiety, and the mass difference of 481 - 285 *m/z* is consistent with the loss of water. Additionally, the ion at *m/z* 451 (ID35) shows a fragment at *m/z* 319, which suggests the presence of a pentoside moiety (132 Da) and was tentatively identified as myricetin pentoside. These three myricetin derivatives, myricetin *O*-sambubioside, myricetin 3-*O*-β-D-galactopyranose and myricetin pentoside were found in *P. graveolens*.

IDs 32, 34, 36, 41, and 49, corresponding to masses 627, 597, 611, 465, 435 and 491 *m/z*, all show a major peak at *m/z* 303. Based on its mass, the fragment at *m/z* 303 was tentatively assigned to the quercetin aglycone ion. The pseudo molecular ion at *m/z* 627 (ID32) showed a fragmentation pattern consistent with the linkage of two glycosyl moieties, which has been described as quercetin 3,4'-diglucoside (Häkkinen and Auriola 1998). The compound with a parent mass of 597 *m/z* (ID34) was identified as querceeti-3-*O*-vicianoside with a score in MzCloud of 79.4%. It has two additional fragments at *m/z* 465 and 435, which could correspond to the loss of a pentose and a hexose, respectively. The ion *m/z* 611 (ID36) has a high MzCloud value of 96.1%. It has been tentatively assigned to rutin based on its mass and fragmentation pattern. The mass difference of 611 - 303 (308 Da) corresponds to the neutral loss of rutinoside. The spectrum obtained for 491 *m/z* (ID49) is similar to the predicted in silico spectrum at -10V for the molecule quercetin 3-*O*-acetyl-rhamnoside in the FooDB database (compound FDB000163 in the database https://foodb.ca/). Overall, the mass 611 *m/z* corresponding to rutin was found in the three *Pelargonium* species, mass 465 (quercetin hexoside) and 435 *m/z* (quercetin pentoside) were identified in *P. graveolens* and *P. zonale*, whereas masses 627 (quercetin 3,4'-diglucoside) and 597 *m/z* (quercetin-3-*O*-vicianoside) were only found in *P. graveolens* and, 491 *m/z* (quercetin 3-*O*-acetyl-rhamnoside) only in *P. zonale*.

The ion with an *m/z* value of 287 is consistently observed as a major ion in IDs 38, 40, 45, and 46. This peak was tentatively identified as the kaempferol product ion. The parent mas 595 *m/z* in *P. zonale* (ID38 and RT = 14.78 min) fragmented mainly in 287 and 449 *m/z*. The difference in mass from 595 to 287 *m/z* (308 Da) may be due to the loss of a glucorhamnoside, and we therefore identified this molecule as kaempferol-glucorhamnoside, which was also identified in the MzCloud database with a score of 91.0%. ID40 with a mass of 595 *m/z* was detected at retention times of 15.36 and 15.95 min in both *P. zonale* and *P. odoratissimum*. The observed fragmentation pattern suggests the presence of a possible kaempferol-glucorhamnoside isomer in both species. In ID45 we found a molecular ion at 449 *m/z*, which was identified in MzCloud with a score of 89.9% as trifolin in *P. graveolens* and *P. odoratissimum*. The fragmentation at 287 *m/z* [M+H-162]+ suggests the presence of a hexoside moiety of 162 Da. Finally, the precursor mass 419 *m/z* (ID46) was identified as juglanin (MzCloud score 91.7%). The loss of 132 uma (pentoside) from 419 to 287 *m/z* is consistent with a pentoside derivative. The mass of 419 *m/z* (juglanin) is found in *P. graveolens* and *P. zonale*.

ID42, detected exclusively in *P. odoratissimum*, showed a molecular ion at 437 *m/z* and was tentatively identified as naringin dihydrochalcone (MzCloud score 87.1%). However, the calculated molecular mass of naringin dihydrochalcone is 582, which is significantly higher than the observed parent ion mass of 437 *m/z*. This discrepancy suggests that the MzCloud identification may be inaccurate and that the actual compound is likely a glucoside of the parent ion. We therefore propose p-phlorizin (molecular mass of 436) as a more plausible candidate for ID42. The rupture of 373 *m/z* (ID43 detected in *P. graveolens*) into two prominent daughter ions at 211 and 193 *m/z*, according to the literature data, corresponds to 4-hydroxy-5-methoxy-trans-melilotoside (Yang et al. 2007). The 625 *m/z* fragment (ID44) at 317 *m/z* is consistent with a loss of rutinoside and rhamnetin product ions with a MzCloud score of 78.1%. Thus, this molecule was tentatively identified as rhamnetin-3-*O*-rutinoside. In addition, the fragment pattern of the parent mass at 275 *m/z* (ID47) is consistent with phloretin (MzCloud score 87.1%). Finally, ID48 was previously identified as *Ov*-NCC-1 (Müller et al. 2011), a compound analogous to Hv-NCC-1, featuring a quasi-molecular ion at *m/z* 679. This compound has a quasimolecular ion at *m/z* 679. It is characterised by the loss of methanol (*m/z* 647) and a ring (*m/z* 522). In general, ions 625 *m/z* (rhamnetin-3-*O*-rutinoside), 275 *m/z* (phloretin), and 679 *m/z* (Ov-NCC-1) were only detected in *P. graveolens.*

**References**

Al-Sayed, Eman, Olli Martiskainen, Sayed H. Seif El-Din, Abdel Nasser A. Sabra, Olfat A. Hammam, and Naglaa M. El-Lakkany. 2015. “Protective Effect of Pelargonium Graveolens against Carbon Tetrachloride-Induced Hepatotoxicity in Mice and Characterization of Its Bioactive Constituents by HPLC-PDA-ESI-MS/MS Analysis.” *Medicinal Chemistry Research* 24(4):1438–48. doi: 10.1007/s00044-014-1218-3.

Callemien, Delphine, and Sonia Collin. 2008. “Use of RP-HPLC-ESI(–)-MS/MS to Differentiate Various Proanthocyanidin Isomers in Lager Beer Extracts.” *Journal of the American Society of Brewing Chemists* 66(2):109–15. doi: 10.1094/ASBCJ-2008-0215-01.

Engström, Marica T., Maija Pälijärvi, Christos Fryganas, John H. Grabber, Irene Mueller-Harvey, and Juha-Pekka Salminen. 2014. “Rapid Qualitative and Quantitative Analyses of Proanthocyanidin Oligomers and Polymers by UPLC-MS/MS.” *Journal of Agricultural and Food Chemistry* 62(15):3390–99. doi: 10.1021/jf500745y.

Fernandez-Soto, Paulina, Diana Celi, Eduardo Tejera, José Miguel Alvarez-Suarez, and António Machado. 2023. “Cinnamomum Sp. and Pelargonium Odoratissimum as the Main Contributors to the Antibacterial Activity of the Medicinal Drink Horchata: A Study Based on the Antibacterial and Chemical Analysis of 21 Plants.” *Molecules* 28(2):693. doi: 10.3390/molecules28020693.

Häkkinen, Sari, and Seppo Auriola. 1998. “High-Performance Liquid Chromatography with Electrospray Ionization Mass Spectrometry and Diode Array Ultraviolet Detection in the Identification of Flavonol Aglycones and Glycosides in Berries.” *Journal of Chromatography A* 829(1–2):91–100. doi: 10.1016/S0021-9673(98)00756-0.

Hokkanen, Juho, Sampo Mattila, Laura Jaakola, Anna Maria Pirttilä, and Ari Tolonen. 2009. “Identification of Phenolic Compounds from Lingonberry (Vaccinium Vitis-Idaea L.), Bilberry (Vaccinium Myrtillus L.) AndHybrid Bilberry (Vaccinium x Intermedium Ruthe L.) Leaves.” *Journal of Agricultural and Food Chemistry* 57(20):9437–47. doi: 10.1021/jf9022542.

Leblanc, André, and Lekha Sleno. 2011. “Atrazine Metabolite Screening in Human Microsomes: Detection of Novel Reactive Metabolites and Glutathione Adducts by LC-MS.” *Chemical Research in Toxicology* 24(3):329–39. doi: 10.1021/tx200008f.

Lin, Long-Ze, Jianghao Sun, Pei Chen, Maria J. Monagas, and James M. Harnly. 2014. “UHPLC-PDA-ESI/HRMS n Profiling Method To Identify and Quantify Oligomeric Proanthocyanidins in Plant Products.” *Journal of Agricultural and Food Chemistry* 62(39):9387–9400. doi: 10.1021/jf501011y.

Liu, Pengzhan, Heikki Kallio, and Baoru Yang. 2014. “Flavonol Glycosides and Other Phenolic Compounds in Buds and Leaves of Different Varieties of Black Currant (Ribes Nigrum L.) and Changes during Growing Season.” *Food Chemistry* 160:180–89. doi: 10.1016/j.foodchem.2014.03.056.

Lozovaya, Vera V., Anatoliy V. Lygin, Olga V. Zernova, Alexander V. Ulanov, Shuxian Li, Glen L. Hartman, and Jack M. Widholm. 2007. “Modification of Phenolic Metabolism in Soybean Hairy Roots through down Regulation of Chalcone Synthase or Isoflavone Synthase.” *Planta* 225(3):665–79. doi: 10.1007/s00425-006-0368-z.

Macz-Pop, Glenda A., Julián C. Rivas-Gonzalo, José J. Pérez-Alonso, and Ana M. González-Paramás. 2006. “Natural Occurrence of Free Anthocyanin Aglycones in Beans (Phaseolus Vulgaris L.).” *Food Chemistry* 94(3):448–56. doi: 10.1016/j.foodchem.2004.11.038.

Müller, Thomas, Sheran Oradu, Demian Ifa, Graham Cooks, and Bernhard Kräutler. 2011. “Direct Plant Tissue Analysis and Imprint Imaging by Desorption Electrospray Ionization Mass Spectrometry.” *Analytical Chemistry* 83(14):5754--5761. doi: https://doi.org/10.1021/ac201123t.

Oven, M., K. Raith, R. H. H. Neubert, T. M. Kutchan, and M. H. Zenk. 2001. “Homo-Phytochelatins Are Synthesized in Response to Cadmium in Azuki Beans.” *Plant Physiology* 126(3):1275–80. doi: 10.1104/pp.126.3.1275.

Overy, David P., David P. Enot, Kathleen Tailliart, Helen Jenkins, David Parker, Manfred Beckmann, and John Draper. 2008. “Explanatory Signal Interpretation and Metabolite Identification Strategies for Nominal Mass FIE-MS Metabolite Fingerprints.” *Nature Protocols* 3(3):471–85. doi: 10.1038/nprot.2007.512.

Ramos-Escudero, Fernando, Celestino Santos-Buelga, José Joaquín Pérez-Alonso, Jaime A. Yáñez, and Montserrat Dueñas. 2010. “HPLC-DAD-ESI/MS Identification of Anthocyanins in Dioscorea Trifida L. Yam Tubers (Purple Sachapapa).” *European Food Research and Technology* 230(5):745–52. doi: 10.1007/s00217-010-1219-5.

Shui, Guanghou, Shih Peng Wong, and Lai Peng Leong. 2004. “Characterization of Antioxidants and Change of Antioxidant Levels during Storage of Manilkara Zapota L.” *Journal of Agricultural and Food Chemistry* 52(26):7834–41. doi: 10.1021/jf0488357.

da Silva, Fátima Lopes, María Teresa Escribano-Bailón, José Joaquín Pérez Alonso, Julián C. Rivas-Gonzalo, and Celestino Santos-Buelga. 2007. “Anthocyanin Pigments in Strawberry.” *Lwt* 40(2):374–82. doi: 10.1016/j.lwt.2005.09.018.

Yang, Li, Norio Nakamura, Masao Hattori, Zhengtao Wang, Annie Bligh, and Luoshan Xu. 2007. “High-Performance Liquid Chromatography-Diode Array Detection/Electrospray Ionization Mass Spectrometry for the Simultaneous Analysis of Cis-, Trans- and Dihydro-2-Glucosyloxycinnamic Acid Derivatives.” *Rapid Communications in Mass Spectrometry* 21:1833–1840. doi: 10.1002/rcm.3025.

Yao, Huina, Ying Peng, and Jiang Zheng. 2016. “Identification of Glutathione and Related Cysteine Conjugates Derived from Reactive Metabolites of Methyleugenol in Rats.” *Chemico-Biological Interactions* 253:143–52. doi: 10.1016/j.cbi.2016.05.006.
