# Supplementary material for: A chemical analysis of the Pelargonium species: P. odoratissimum, P. graveolens, and P. zonale identifies secondary metabolites with activity against gram-positive bacteria with multidrug-resistance
Source: PLoS One. 2024 Jul 10;19(7):e0306637. doi: 10.1371/journal.pone.0306637 (PMC11236107; doi:10.1371/journal.pone.0306637)
Supplement: S2 Table — (DOCX) [file pone.0306637.s004.docx]

**Supplementary Table 2.** Biofilm inhibition and eradication of bacteria of clinical importance by *P. graveolens*, *P. odoratissimum*, and *P. zonale*.

|  |  | | **Biofilm inhibition/ eradication assays** | | | |  |
| --- | --- | --- | --- | --- | --- | --- | --- |
| **Plant**  **scientific name** | Bacterial Strains | Type | Biofilm inhibition (%) | *p*-values | Biofilm eradication (%) | *p*-values | |
| ***Pelargonium graveolens***  **1000** **µg/mL** | *S. aureus* ATCC 25923 | Methanol-water Control | 57.3 ± 3.9 | 0.003 ^a^ | 57.8 ± 3.1 | 0.0007 ^a^ | |
|  |  | 1X MIC | 42.9 ± 6.8 | 0.013 ^b^ | 18.9 ± 14.9 | 0.002 ^b^ | |
|  |  | 2X MIC | 46.0 ± 4.6 | 0.014 ^b^ | 42.8 ± 12.3 | 0.007 ^b^ | |
|  | *E. faecalis* ATCC 29212 | Methanol-water Control | 25.5 ± 4.4 | 0.0009 ^a^ | 66.9 ± 3.8 | 0.001 ^a^ | |
|  |  | 1X MIC | 22.5 ± 8.2 | 0.493 ^b^ | 37.6 ± 3.4 | 0.0007 ^b^ | |
|  |  | 2X MIC | 31.7 ± 4.3 | 0.036 ^b^ | 64.8 ± 1.5 | 0.316 ^b^ | |
|  | *E. faecalis* INSPI 032 | Methanol-water Control | 8.9 ± 3.2 | 0.0009 ^a^ | 35.8 ± 12.1 | 0.0002 ^a^ | |
|  |  | 1X MIC | 20.4 ± 2.8 | 0.0006 ^b^ | 23.9 ± 7.0 | 0.0420 ^b^ | |
|  |  | 2X MIC | 31.5 ± 7.9 | 0.0004 ^b^ | 28.2 ± 11.9 | 0.0182 ^b^ | |
| ***Pelargonium odoratissimum***  **1000** **µg/mL** | *S. aureus* ATCC 25923 | Methanol-water Control | 57.3 ± 3.9 | 0.003 ^a^ | 57.8 ± 3.1 | 0.0007 ^a^ | |
|  |  | 1X MIC | 42.0 ± 1.2 | 0.007 ^b^ | 38.3 ± 12.6 | 0.004 ^b^ | |
|  |  | 2X MIC | 42.6 ± 2.1 | 0.010 ^b^ | 51.9 ± 8.5 | 0.181 ^b^ | |
| ***Pelargonium zonale***  **250** **µg/mL** | *S. aureus* ATCC 25923 | Methanol-water Control | 57.3 ± 3.9 | 0.003 ^a^ | 57.8 ± 3.1 | 0.0007 ^a^ | |
|  |  | 1X MIC | 47.5 ± 3.9 | 0.013 ^b^ | 31.6 ± 14.6 | 0.002 ^b^ | |
|  |  | 2X MIC | 51.3 ± 4.2 | 0.062 ^b^ | 37.7 ± 16.4 | 0.010 ^b^ | |
|  | *S. aureus* MRSA 333 | Methanol-water Control | 31.1 ± 3.3 | 0.006 ^a^ | 26.8 ± 5.9 | 0.004 ^a^ | |
|  |  | 1X MIC | 20.3 ± 1.3 | 0.005 ^b^ | 23.1 ± 9.4 | 0.408 ^b^ | |
|  |  | 2X MIC | 30.3 ± 2.2 | 0.666 ^b^ | 34.0 ± 9.7 | 0.068 ^b^ | |
| ***Pelargonium zonale***  **1000** **µg/mL** | *E. faecalis* INSPI 032 | Methanol-water Control | 8.9 ± 3.2 | 0.0009 ^a^ | 35.8 ± 12.1 | 0.0002 ^a^ | |
|  |  | 1X MIC | 30.3 ± 5.4 | 0.0002 ^b^ | 8.7 ±9.7 | 0.0007 ^b^ | |
|  |  | 2X MIC | 41.8 ± 9.0 | 0.0004 ^b^ | 29.6 ± 10.9 | 0.193 ^b^ | |

MIC: Minimum inhibitory concentration, which is indicated in each plant extract information. Biofilm formation values of methanol-water controls and samples (1X and 2X MIC) were calculated as the percentage of bacteria biofilm formation through the optical density comparison between methanol-water controls/samples and bacterial growth in only medium culture (positive control). The positive controls were considered 100% when compared to the methanol-water control and samples in the assays. Statistical analysis (*p*-values) was performed using non-parametric Wilcoxon test (95% confidence interval) to compare biofilm formation values. ^a^ *p*-value obtained when comparing positive controls and methanol-water controls. ^b^ *p*-value obtained when comparing methanol-water controls and sample
