# Supplementary material for: A chemical analysis of the Pelargonium species: P. odoratissimum, P. graveolens, and P. zonale identifies secondary metabolites with activity against gram-positive bacteria with multidrug-resistance
Source: PLoS One. 2024 Jul 10;19(7):e0306637. doi: 10.1371/journal.pone.0306637 (PMC11236107; doi:10.1371/journal.pone.0306637)
Supplement: S3 Table — (DOCX) [file pone.0306637.s005.docx]

**Supplementary Table 3.** Tentative identification of metabolites identified in fractions 1 – 4 in *P. zonale* in positive ionization mode.

| **Peak** | **ID** | **RT (min)** | **Parent**  **mass**  ***m/z*** | **Main MS/MS products (*m/z*)** | **Tentative identification** | **Resource** |
| --- | --- | --- | --- | --- | --- | --- |
| 1 | 1 | 4.37 | 192  [M+H]^+^ | 122(100) 120(95) 82(74) 121(48) 123(32) 174(19) 183(17) 160(15) 83(11) 130(9)175(8) 70(6) 84(6) 161(6) 146(7) | UI |  |
| 2 | 2 | 4.89 | 381  [M+H]^+^ | 201(80) 213(5) 219(100) | Sucrose | (Overy et al. 2008) |
|  | 3 | 4.91 | 147 | 147(100) | UI |  |
| 3 | 4 | 5.14 | 116 | 116(100) | UI |  |
| 4 | 5 | 5.26 | 258 | 104(100) 240(8) | UI |  |
| 5 | 6 | 5.77 | 678 | 336(16) 354(100) 498(10) 516(79) | UI |  |
|  | 7 | 6.00 | 132 |  | UI |  |
| 6 | 8 | 6.07 | 595  [M]+ | 271(95) 433(100) | Pelargonidin 3,5-diglucoside |  |
|  | 9 | 6.11 | 166 | 166(100) | UI |  |
| 7 | 10 | 6.23 | 205  [M+H]^+^ | 188(100) 159(1)4 | DL-tryptophan | 91.4% |
| 8 | 11 | 6.69 | 487 | 143(8) 144(86) 161(100) 327(71) 469(6) | UI |  |
| 9 | 12 | 7.08 | 227 | 85(8) 167(5) 191(42) 209(100) | UI |  |
| 10 | 13 | 7.98 | 185 | 153(100) | UI |  |
| 11 | 14 | 8.28 | 339  [M+H]^+^ | 119(8) 147(100) 321(11) | Coumaroylquinic acid isomer 1 | 80.3% |
| 12 | 15 | 8.64 | 619  [M-OH]^+^ | 233(5) 237(7) 261(12) 279(14)  297(6) 305(12) 431(6) 449(100)  467(14) | 1,2,6-Trigalloylglucose | 95.0% |
| 13 | 16 | 8.98 | 369  [M+H]^+^ | 145(10) 177(100) | 4-*O-*feruloyl-D-quinic acid | 84.6% |
| 14 | 17 | 9.16 | 339  [M+H]^+^ | 119(8) 147(100) 290(5) 321(11) | Coumaroylquinic acid isomer 2 | 80.8% |
| 15 | 18 | 9.75 | 611  [M+H]^+^ | 303(100) 449(6) 465(28) | Rutin | 96.1% |
| 16 | 19 | 10.02 | 771  [M-OH]^+^ | 233(16) 243(9) 261(56) 279(100)  305(72) 413(12) 431(34) 449(21)  583(6) 601(13) 619(13) | Tetraglalloyglucopyranose | 74.7% |
| 17 | 20 | 10.66 | 465  [M+H]^+^ | 303(100) | Quercetin hexoside | 83.7% |
| 18 | 21 | 11.04 | 595  [M+H]^+^ | 271(8) 287(100) 433(15) 449(41) | Kaempferol-glucorhamnoside | 85.9% |
| 19 | 22 | 11.3 | 268 | 84(100) 227(5) | UI |  |
| 20 | 23 | 11.51 | 609  [M+H]^+^ | 303(100) 345(15) 411(5) 573(8) 591(13) | Quercetin 3-[6''-(3-hydroxy-3-methylglutaryl)galactoside] | 91.0% |
|  | 24 | 11.68 | 695 | 488(7) 506(9) 520(17) 538(5) 627(9) 641(6) 645(77) 659(6) 663(11) 677(100) | UI |  |
| 21 | 25 | 11.64 | 595  [M+H]^+^ | 287(100) 449(20) | Kaempferol-glucorhamnoside (isomer) | 97.0% |
| 22 | 26 | 11.91 | 923  [M-OH]^+^ | 279(11) 305(100) 413(27) 431(13) 431(67) 456(9) 457(22) 565(5) 583(8) 583(21) 601(14) 735(10) 753(12) 771(33) | Pentaglalloyglucopyranose |  |
| 23 | 27 | 12.20 | 435  [M+H]^+^ | 303(100) | Quercetin pentoside | 97.3% |
| 24 | 28 | 12.69 | 449  [M+H]^+^ | 287(100) | Trifolin | 91.7% |
|  | 29 | 13.05 | 1075  [M-OH]^+^ | 395(5) 413(18) 431(12) 431(44) 457(60) 565(11) 583(21) 601(10) 609(18) 717(5) 735(26) 753(16) 771(12) 887(13) 905(16) 923(100) | Hexaglalloyglucopyranose | See comment |
| 25 | 30 | 13.20 | 1227  [M-OH]^+^ | 583(20) 735(29) 923(20) 1075(100) | Heptaglalloyglucopyranose | See comment |
|  | 31 | 13.28 | 593 | 287(100) 329(12) 395(8) 557(9) 575(21) | UI | See comment |
|  | 32 | 13.33 | 419  [M+H]^+^ | 287(100) 383(5) | Juglanin | 91.7% |
| 26 | 33 | 13.89 | 419  [M+H]^+^ | 287(100) 383(5) 401(15) | Juglanin (isomer) | 87.2% |
| 27 | 34 | 14.01 | 679 | 490(6) 504(7) 611(7) 629(9) 629(53) 643(7) 647(26) 661(100) | UI |  |
| 28 | 35 | 14.23 | 433  [M+H]^+^ | 271(21) 287(100) 397(5) | Kaempferol-3-*O*-β-rhamnoside | 79.8% |
| 29 | 36 | 14.84 | 491 | 287(100) 395(7) 473(5) | UI | See comments |
| 30 | 37 | 15.11 | 515 | 161(11) 175(100) 193(22) 318(5) 336(36) 353(34) 498(18) | UI |  |
| 31 | 38 | 16.22 | 475 | 457(100) 439(15) 287(15) 312(5) 275(5) | UI |  |
| 32 | 39 | 16.38 | 287  [M+H]^+^ | 121(16) 133(12) 135(15) 145(12)  153(100) 161(11) 165(53) 197(12)  213(48) 231(25) 241(72) 245(18) 258(28) 259(20) 269(17) | Kaempferol | 89.7% |

Unidentified compound (UI). The percentage indicates the score reported in the MzCloud database.

A new mass identified in fractions 1 to 4 was the compound at 619 *m/z* (ID15, Table 5) which was initially identified in MzCloud as 1,2,6-tri-*O*-galloylglucose, a type of gallotannin, with a score of 95%. However, this compound has a molar mass of 636. We therefore proposed that it forms an adduct as [M-OH]+ with an *m/z* of 619. Other gallotannins were also detected in the four fractions at *m/z* 771 (MzMine score 74.7), *m/z* 923, *m/z* 1075, and *m/z* 1227, with retention times of 10.02, 11.91, 13.05, and 13.20 minutes, respectively. These peaks showed a consistent and consecutive mass increase of 152 Da, which is associated with the galloyl moiety present in gallotannins (Wang et al. 2016). According to Da Silva et al., (2011), these series of peaks are also represented by the adduct [M - OH]+ and correspond to tetra-, penta, hexa-, hepta-, and octagalloylglucopyranose, respectively (da Silva et al. 2007).

Interestingly, the mass 339 *m/z* appears at RT= 8.28 (ID14) and RT = 9.16 (ID17). This parent mass was previously identified as coumaroylquinic acid and the two presences at different retention times and remarkably similar rupture, suggesting possible isomers. The ID16 (RT = 8.98 min) showed the [M+H]+ ion at *m/z* 369. The fragmentation pattern gave the major ion at *m/z* 177 [ferulic acid-H2O+H]+, corresponding to the loss of the quinic acid moiety (192 Da), and a low-intensity ion at *m/z* 145 [ferulic acid-H2O-CH3OH+H]+ (Ren et al. 2013). The molecular ion at *m/z* 609 (ID23) was detected and identified as quercetin 3-[6'-(3-hydroxy-3-methylglutaryl)galactoside] in MzCloud with a score of 91%. The mass difference of 609 - 303 *m/z* (306 Da) is attributed to the loss of an acetylated hexoside with 3-hydroxy-3-methylglutaric (HMG) acid (Porter et al. 2012). It is interesting to note that IDs 31 and 36 have a major ion at 287 *m/z*, which suggests that they may also contain a kaempferol aglycone. However, no information was found about the fragmentation patterns of these ions in the databases and the literature. Despite a throughout analysis, 17 masses from the four fractions remained unidentified. In some instances, this was due to the presence of a single peak corresponding to the parent ion in the fragmentation pattern (IDs 3, 4, and 9). In other cases, the mass exhibited no fragmentation pattern at all (ID7). The fragmentation patterns of the remaining unidentified masses were not found in either databases or literature.

**References**

Overy, David P., David P. Enot, Kathleen Tailliart, Helen Jenkins, David Parker, Manfred Beckmann, and John Draper. 2008. “Explanatory Signal Interpretation and Metabolite Identification Strategies for Nominal Mass FIE-MS Metabolite Fingerprints.” *Nature Protocols* 3(3):471–85. doi: 10.1038/nprot.2007.512.

Porter, Elaine A., Alexander A. Van Den Bos, Geoffrey C. Kite, Nigel C. Veitch, and Monique S. J. Simmonds. 2012. “Flavonol Glycosides Acylated with 3-Hydroxy-3-Methylglutaric Acid as Systematic Characters in Rosa.” *Phytochemistry* 81:90–96. doi: 10.1016/j.phytochem.2012.05.006.

Ren, Qiang, Caisheng Wu, Yan Ren, and Jinlan Zhang. 2013. “Characterization and Identification of the Chemical Constituents from Tartary Buckwheat (Fagopyrum Tataricum Gaertn) by High Performance Liquid Chromatography/Photodiode Array Detector/Linear Ion Trap FTICR Hybrid Mass Spectrometry.” *Food Chemistry* 136(3–4):1377–89. doi: 10.1016/j.foodchem.2012.09.052.

da Silva, Fátima Lopes, María Teresa Escribano-Bailón, José Joaquín Pérez Alonso, Julián C. Rivas-Gonzalo, and Celestino Santos-Buelga. 2007. “Anthocyanin Pigments in Strawberry.” *Lwt* 40(2):374–82. doi: 10.1016/j.lwt.2005.09.018.

Wang, Yuehua, Jinyan Zhu, Xianjun Meng, Suwen Liu, Jingjing Mu, and Chong Ning. 2016. “Comparison of Polyphenol, Anthocyanin and Antioxidant Capacity in Four Varieties of Lonicera Caerulea Berry Extracts.” *Food Chemistry* 197:522–29. doi: 10.1016/j.foodchem.2015.11.006.
